# Locality and Word Order in Active Dependency Formation in Bangla

**DOI:** 10.3389/fpsyg.2016.01235

**Published:** 2016-08-25

**Authors:** Dustin A. Chacón, Mashrur Imtiaz, Shirsho Dasgupta, Sikder M. Murshed, Mina Dan, Colin Phillips

**Affiliations:** ^1^Department of Linguistics, University of Maryland, College ParkCollege Park, MD, USA; ^2^Department of Linguistics, University of MinnesotaMinneapolis, MN, USA; ^3^Department of Linguistics, University of DhakaDhaka, Bangladesh; ^4^Department of Linguistics, University of CalcuttaKolkata, India

**Keywords:** filler-gap dependencies, locality, Bangla, sentence processing, islands

## Abstract

Research on filler-gap dependencies has revealed that there are constraints on possible gap sites, and that real-time sentence processing is sensitive to these constraints. This work has shown that comprehenders have preferences for potential gap sites, and immediately detect when these preferences are not met. However, neither the mechanisms that select preferred gap sites nor the mechanisms used to detect whether these preferences are met are well-understood. In this paper, we report on three experiments in Bangla, a language in which gaps may occur in either a pre-verbal embedded clause or a post-verbal embedded clause. This word order variation allows us to manipulate whether the first gap linearly available is contained in the same clause as the filler, which allows us to dissociate *structural locality* from *linear locality*. In Experiment 1, an untimed ambiguity resolution task, we found a global bias to resolve a filler-gap dependency with the first gap linearly available, regardless of structural hierarchy. In Experiments 2 and 3, which use the filled-gap paradigm, we found sensitivity to disruption only when the blocked gap site is both structurally and linearly local, i.e., the filler and the gap site are contained in the same clause. This suggests that comprehenders may not show sensitivity to the disruption of all preferred gap resolutions.

## Introduction

The formation of linguistic dependencies is subject to a wide variety of constraints. Some constraints are conditions on grammatical well-formedness, whereas others define the interpretations that are preferred in real-time sentence processing. Locality constraints on filler-gap dependencies are one particularly well-studied example of both constraint types. Some locality constraints distinguish acceptable filler-gap dependencies from unacceptable filler-gap dependencies, as long recognized by syntacticians (Ross, 1967; Huang, 1982; Rizzi, 1982, 1990, 2013; Chomsky, 1986; Rudin, 1988; Lasnik and Saito, 1992; Manzini, 1992; Szabolcsi and den Dikken, 1999; Boeckx, 2008). For instance, the filler-gap dependency in (1a) between *who* and the position in which it is interpreted (marked as ___) is judged acceptable, in contrast with the sentence in (1b). This is because filler-gap dependencies may not cross into clauses (marked S′) in the subject position of another clause (this violates the *sentential subject constraint* and the *complex noun phrase constraint*, Ross, 1967). Constraints on acceptable filler-gap dependencies are called *island constraints*.

(1) a. I know **who** it surprised Dale [_S′_ that Sarah saw ___].
b. ^*^I know **who** [_S′_ that Sarah saw ___] surprised Dale.

Other locality constraints determine which gap sites are preferred when multiple possibilities are available. In on-line tasks, this manifests as a preference for early resolution, a process called *active dependency formation* (Fodor, 1978; Crain and Fodor, 1985; Stowe, 1986; Frazier, 1987; Frazier and Flores d'Arcais, 1989). For instance, Stowe (1986) observed longer reading times at the direct object *us* in (2a) compared to the control sentence in (2b), which lacks a filler-gap dependency. This increase in reading times, called the *filled-gap effect*, suggests that readers make an early commitment to resolve *who* as the direct object of *bring* before it is clear whether there is a direct object gap. Encountering the direct object pronoun *us* then triggers a reanalysis process, leading to an increase in processing difficulty.

(2) a. My brother wanted to know **who** Ruth would bring us home to ___ at Christmas.
b. My brother wanted to know if Ruth would bring us home to somebody at Christmas.

There has been much interest in determining whether these two types of constraints are the same, following from some independently motivated restrictions on linguistic processes, e.g., restrictions on memory capacity (Deane, 1991; Pritchett, 1992; Kluender and Kutas, 1993; Kluender, 1998, 2004; Hofmeister and Sag, 2010; for discussion see Phillips, 2013). Explaining island phenomena as a consequence of resource limitation has the potential to radically simplify grammatical theories.

If island constraints are indeed reducible to constraints on preferred gap sites, then both sets of constraints should be sensitive to the same properties of the linguistic representation being computed. In other words, the notion of “local” that is relevant should be the same. It is relatively uncontroversial that island constraints are defined in terms of formal linguistic structure, either hierarchical syntactic relations (Ross, 1967; Chomsky, 1981, 1986; Huang, 1982; Rizzi, 1990; Lasnik and Saito, 1992; for review, see Rizzi, 2013), or semantic/pragmatic relations (Erteschik-Shir, 1973; Kuno, 1976; Szabolcsi and Zwarts, 1993; Truswell, 2007; Ambridge and Goldberg, 2008; Abrusán, 2011a,b). However, it is unclear what notion of locality is relevant for determining preferred gap sites. For instance, the direct object position of *bring* in (2b) may be preferred, because fewer nodes separate this gap site from the filler compared to other potential forthcoming gap sites, i.e., there is an additional PP node separating the filler and prepositional object gap site, illustrated in (3). To construct the direct object gap, the comprehender needs to postulate a less articulated structure (a verb phrase and an object position) than in alternative analyses (a verb phrase, plus dependents on this verb phrase, such as a prepositional phrase, and an object position). Alternatively, the direct object position may be preferred because it is the first position that is linearly available. That is, the locality constraints on preferred gap sites may be defined in terms of *structural locality* or *linear locality*. If the constraints on preferred gap sites are sensitive to linear locality, then this motivates maintaining a distinction between island constraints and locality constraints on preferred gap sites.

(3) My brother wanted to know
**who** [_S_ Ruth would [_VP_ bring us home **[_PP_** to ___**]** at Christmas]]]

Most research on filler-gap dependency processing cannot decide among these hypotheses, because most studies are conducted on languages like English, where structural and linear locality converge, as illustrated above. However, previous work on Japanese, a language with different word order properties than English, suggests that these constraints are dissociated (Aoshima et al., 2004; Yoshida, 2006; Omaki et al., 2013). This is discussed in more detail in Section Locality in Filler-Gap Dependencies.

In this paper, we report on three experiments in Bangla (Bengali) that further investigate locality constraints on preferred gap sites. Bangla is a valuable language for this purpose, because embedded clauses may either precede or follow the embedding verb, as shown in (4) and (5). Additionally, Bangla allows filler-gap dependencies with *wh*-phrases. These filler-gap dependencies may resolve in either the main clause, or an embedded clause on either side of the main verb, as shown in (6) and (7). This allows us to manipulate whether the first gap site is structurally local or distant within the same language, which allows a within-language comparison of the influence of word order on filler-gap dependency processing, which has previously only been conducted in a cross-language fashion (Omaki et al., 2013).

(4) raj bollo [_S′_ še ašbe ]
  Raj said he come.fut
  “Raj said that he will come.”

(5) raj [_S′_ še ašbe ] bollo
  Raj he come.fut said
  “Raj said that he will come.”

(6) raj **kɔkhon** ___ bollo [_S′_ še ___ ašbe ]
  raj when said he come.fut
  “When did Raj say ___ that he will come ___ ?”

(7) raj **kɔkhon** [_S′_ še ___ ašbe ] ___ bollo
  raj when he come.fut said
  “When did Raj say ___ that he will come ___ ?”

Experiment 1 was a within-language replication of the cross-language findings from Omaki et al. (2013). In Experiment 1, we investigated how ambiguous filler-gap dependencies like (6) and (7) are resolved using an off-line ambiguity resolution task. This task allows us to probe for preferences directly, instead of relying on an indirect measure, such as increased reading times indicating detection of an unexpected parse. We found that filler-gap dependencies are resolved with the first position linearly available across word orders. In main verb first word orders as in (6), the filler-gap dependency was resolved with the main verb. In embedded verb first word orders like (7), it was resolved with the embedded verb.

In Experiment 2, we investigated the preference for linearly local gap sites in an on-line, filled-gap paradigm task. This task provides a more standard measure of disruption in moment-by-moment sentence comprehension, and thus it can be used to determine the time course of active dependency formation. Like Experiment 1, we leveraged the flexible word order of Bangla to manipulate whether the first available gap site was in the same clause as the filler or in an embedded clause. We found a filled-gap effect when resolution with the first gap site was blocked in main verb first word orders like (6), where structural locality and linear locality aligned, but not in embedded verb first word orders like (7). In other words, there was only detection of a blocked filler-gap resolution when the gap site was both structurally local and linearly local, but not when this position was structurally distant. The comparison between Experiments 1 and 2 suggests a contrast between gap site preferences and sensitivity to disruption.

The apparent mismatch in Experiments 1 and 2 may be due to the on-line/off-line contrast between the two experiments, or to the ambiguity resolution/filled-gap paradigm difference. In Experiment 3, we diagnosed the cause of this mismatch. Experiment 3 was an off-line acceptability judgment task, like Experiment 1, that used the filled-gap paradigm, like Experiment 2. We again only found evidence that comprehenders detected a filled-gap when the filler-gap dependency was blocked from resolving with a structurally local and linearly local position, as in Experiment 2. This suggests that the contrast between locality preferences and sensitivity to disruption for embedded verb first word orders in Experiments 1 and 2 was not due to the off-line/on-line contrast, but rather the specific mechanisms underlying filled-gap detection.

## Locality in filler-gap dependencies

There is substantial evidence that shorter filler-gap dependencies are preferred to longer filler-gap dependencies. For instance, Frazier and Clifton (1989) found that reading times were increased for sentences containing filler-gap dependencies spanning multiple clauses compared to controls (see also Kluender and Kutas, 1993; Dickey, 1996; Kluender, 1998). This bias against longer filler-gap dependencies is also reflected in offline acceptability judgments, where sentences containing filler-gap dependencies spanning multiple clauses are rated lower than sentences with shorter filler-gap dependencies (Phillips et al., 2005; Alexopoulou and Keller, 2007; Sprouse et al., 2012).

Online studies show that the preference for shorter filler-gap dependencies manifests as a preference for early resolution. For instance, the filled-gap effect discussed in Section Introduction demonstrates that blocking an early filler-gap dependency resolution triggers a costly reanalysis process (Crain and Fodor, 1985; Stowe, 1986; Lee, 2004). Converging evidence comes from the plausibility mismatch paradigm (Garnsey et al., 1989; Traxler and Pickering, 1996). For instance, in a series of eye-tracking experiments, Traxler and Pickering (1996) observed that gaze times increased on the verb *wrote* in (8b) compared to (8a).

(8) a. We like **the book** that the author wrote unceasingly and with great dedication about ___ while waiting for a contract.
b. We like **the city** that the author wrote unceasingly and with great dedication about ___ while waiting for a contract.

This suggests that *the city* was first interpreted as the object of *wrote*. Comprehenders could then detect that the early gap commitment yields an implausible interpretation. Then, they rejected this commitment, and searched for a different gap, yielding a reanalysis cost. Thus, like we argued for the filled-gap effect, the plausibility mismatch effect illustrates not only early commitment to a local gap, but also sensitivity to disruption when this position is unavailable. Other converging evidence for active dependency formation comes from EEG studies (Garnsey et al., 1989; Kaan et al., 2000; Phillips et al., 2005), the “stops making sense” task (Tanenhaus et al., 1985; Boland et al., 1995), cross-modal lexical priming (Nicol and Swinney, 1989; Nicol et al., 1994), and “visual world” eye-tracking (Sussman and Sedivy, 2003).

This bias toward early filler-gap dependency resolution in real-time behavior and toward shorter dependencies in offline judgments is commonly attributed to resource limitations. For instance, unintegrated fillers may require memory resources to be actively maintained (Jackendoff and Culicover, 1971; Wanner and Maratsos, 1978). Alternatively, longer dependencies in general may be more costly, leading to a dispreference for longer filler-gap dependencies (Gibson, 1998; Hawkins, 2004). Other analyses contend that longer filler-gap dependencies may cause increased processing difficulty because the filler must be retrieved from memory at the gap site, which may be costly and error-prone in the case of longer dependencies (McElree, 2006; Wagers and Phillips, 2014). Lastly, more local gaps may be preferred because comprehenders attempt to resolve as many grammatical requirements as early as possible (Pritchett, 1992; Weinberg, 1992; Altmann and Kamide, 1999; Sedivy et al., 1999; Aoshima et al., 2004; Wagers and Phillips, 2009). These accounts all imply that the comprehender should minimize filler-gap dependency length in order to optimize resource usage. However, these accounts make no commitment as to whether linear locality or structural locality are relevant in selecting preferred gap sites.

Island constraints, in contrast, are typically described in structural terms. Island constraints are restrictions on possible filler-gap dependencies, with several illustrated in

(9) a. Relative Clause Island:
  ^*^ **Who** did Dale comfort [_NP_ the woman that [_S_ saw ___ ?]]
b. Whether Island:
  ^*^ **Who** did Dale wonder [whether Bob frightened ___ ?]
c. *Wh*-Island:
  ^*^ **Who** did Dale say [who saw ___ behind Laura's bed?]
d. Subject Island:
  ^*^ **Who** did [the fact that Sarah saw ___] surprise Dale?
e. Adjunct Island:
  ^*^ **Who** did Dale ruminate [while Harry interrogated ___ ?]
f. Coordinate Structure Constraint:
  ^*^ **Who** did [Dale suspect ___ and Harry interrogate Leland?]
g. Factive Island:
  ^*^ **Why** did Dale remember [that Ben was suspicious ___?]

Island constraints have long been studied in theoretical linguistics, where they typically are characterized as constraints on well-formed linguistic representations, either as formal syntactic constraints (Ross, 1967; Chomsky, 1977, 1981, 1986; Huang, 1982; Rizzi, 1990, 2013; Lasnik and Saito, 1992), or as constraints on well-formed and felicitous semantic/pragmatic forms (Erteschik-Shir, 1973; Kuno, 1976; Szabolcsi and Zwarts, 1993; Truswell, 2007; Ambridge and Goldberg, 2008; Abrusán, 2011a,b). As such, island constraints are typically defined over the hierarchical structure of the sentence, or the formal relations between the words and phrases. This can be demonstrated with pairs like (10), repeated from (1), in which the filler-gap dependency that spans fewer words is dispreferred to a filler-gap dependency that spans more words. This contrast can be characterized as a formal constraint against gaps in subject clauses, but not extraposed clauses (Ross, 1967).

(10) a. I know **who** it surprised Dale [_S′_ that Sarah saw ___] ?
b. ^*^ I know **who** [_S′_ that Sarah saw ___] surprised Dale?

Island constraints are observed to be robust in both off-line and on-line measures. Off-line acceptability judgments show that speakers give low ratings to sentences with island violations (Sobin, 1987; Cowart, 1996, 2003; Alexopoulou and Keller, 2007; Heestand et al., 2011; Sprouse et al., 2012). Additionally, the effects of active dependency formation typically disappear in island constructions. There are no filled-gap effects or plausibility mismatch effects inside island contexts (Stowe, 1986; Bourdages, 1992; Traxler and Pickering, 1996). Similarly, results from EEG studies (Neville et al., 1991; Kluender and Kutas, 1993; McKinnon and Osterhout, 1996) and speed-accuracy tradeoff studies (McElree and Griffith, 1998) suggest that comprehenders immediately detect island boundaries. The rapid application of island constraints can be explained in theories of sentence processing that posit rapid and faithful use of grammatical constraints (e.g., Lewis and Phillips, 2015) or theories that posit that representations with gap sites inside island contexts are too costly to represent (Gibson, 1998; Hawkins, 2004).

Some data suggests the constraints on preferred gaps should be dissociated from island constraints (Phillips, 2006; Wagers and Phillips, 2009; Sprouse et al., 2012; Yoshida et al., 2014). Other findings imply that constraints on preferred gap sites are defined in terms of linear locality, unlike island constraints which are defined in terms of structural locality. These findings come from Japanese, a language in which embedded clauses precede the main verb, meaning that in multi-clause sentences structural positions that are linearly closer may be structurally more distant. This makes it possible to dissociate structural locality and linear locality. Japanese speakers prefer to resolve filler-gap dependencies in embedded clauses, likely because this is the first position linearly available. For instance, Aoshima et al. (2004) found filled-gap effects for sentences like (11), in which the fronted dative phrase *dono-syain-ni* “which employee-dat” was blocked from resolving with the embedded clause because of the case-matched noun phrase *kacyoo-ni* “assistant manager-dat” (see also Yoshida, 2006). Similarly, Omaki et al. (2013) showed that speakers of Japanese interpreted an ambiguously fronted *wh*-phrase, as in (12), with the embedded clause in a Question after Story task, a task that provides an untimed measure of how speakers prefer to interpret ambiguous questions (de Villiers et al., 1990). This shows that in off-line measures of gap location preferences and on-line measures of filled-gap detection, Japanese speakers prefer a linearly local resolution.

(11) **Dono-syain-ni** senmu-wa
  which employee-dat managing director-top
  [syacyoo-ga kaigi-de
  president-nom meeting-at
  kacyoo-ni syookyuu-o yakusoku-sita-to]
  assistant manager-dat raise-acc promised-declc
  iimasita-ka?
  told-q?
  “**Which employee** did the managing director tell ___ that the president promised a raise to the assistant manager at the meeting?)”

(12) **Doko-de** Yukiko-chan-wa [choucho-o
  where-at Yukiko-dim-top butterfly-acc
  tsukumaeru-to] itteta-no?
  catch-declc was telling-q?
  “Where did Yukiko say that she will catch butterflies?”

In this paper, we further investigate this generalization in Bangla, a language with variable word order that permits us to manipulate whether the most linearly local potential gap site is within the same clause as the fronted filler (i.e., structurally local), or in an embedded clause (i.e., structurally non-local). In Section Grammatical Properties of Bangla, we describe the relevant properties of Bangla syntax. In Sections Experiment 1–General Discussion we describe the results of three experiments on Bangla filler-gap dependency processing.

## Grammatical properties of Bangla

Bangla is a language spoken primarily in Bangladesh and the eastern Indian state of West Bengal, with approximately 180 million speakers worldwide (Lewis et al., 2015). Bangla is in the Eastern Zone of the Indo-Aryan branch of the Indo-European language family. Due to its contact with multiple linguistic areas, Bangla has many properties typical of northern Indo-Aryan, Dravidian, and Southeast Asian languages. For more complete descriptions of the language, see Thompson (2010) and David (2015).

Embedded clauses in Bangla may either precede or follow an embedding verb, shown in (13). Post-verbal embedded clauses may be introduced with the complementizer *je*, shown in (14a). Pre-verbal embedded clauses may appear with the complementizer *bole* at the end of the clause, shown in (14b), or with *je* in a clause-internal position, shown in (14c). Dasgupta (2007) describes the clause-internal *je* as an “anchor,” which may be a distinct lexical category. Examples are taken from Bayer (1996).

(13)    a. še bollo ora ašbe
                he said    they come.fut
            b. še ora ašbe    bollo
                  he they come.fut said
                ‘He said that they will come’

(14)   a. chele-ṭa bollo [_S′_ je tar    baba   ašbe       ]
               boy-cl said    that his father come.fut
          b. chele-ṭa [_S′_ tar baba ašbe    bole ] bollo
                boy- cl    his father come.fut that   said
          c. chele-ṭa [_S′_ tar baba    je    ašbe       ] bollo
                boy- cl    his father that come.fut said
               ‘The boy said that his father will come'

These constructions are used in similar contexts, although there are subtle syntactic and semantic differences that we leave aside (for discussion see Bal, 1990 on related constructions in Oriya, and Bayer, 1996, 1999, 2001; Simpson and Bhattacharya, 2000, 2003).

Case-marking is often an important cue in detecting clause boundaries in head-final languages. For example, Japanese speakers use nominative-marked noun phrases to detect the beginning of embedded clauses (Miyamoto, 2002). We assume that Bangla speakers do the same, although we have not directly tested this. Bangla has four cases—nominative, accusative, genitive, and oblique. The first three cases are clearly marked in the pronoun system, e.g., š*e* “3sg.nom,” *take* “3sg.acc,” and *tar* “3sg.gen.” Thus, in (13b), the comprehender can detect the embedded clause, because *ora* “3pl.nom” is a clearly nominative-marked pronoun, as is š*e* “3sg.nom.” For other noun phrases, nominative case is left unmarked, and the accusative case morpheme (*-ke*) is reserved for animate objects or specific inanimate objects. In (14b–14c), a comprehender can detect the embedded clause at *baba*, “father.” This is because *baba* “father” is an animate noun that is not marked with an overt accusative, genitive, or oblique morpheme. Thus, it must be nominative. Given that there was a previous nominative noun phrase (*chele-ṭa* “the boy”), the comprehender should postulate an embedded clause here, as well.

Like English and Japanese, Bangla also permits unbounded filler-gap dependencies. Gaps may either occur in pre-verbal or post-verbal embedded clauses. Extraction from a post-verbal clause is shown in (15), adapted from Simpson and Bhattacharya (2003). In (15a), the noun phrase *hæmleṭ* “Hamlet” is interpreted as the direct object of the verb *poṛeche* “read.” In (15b) and (15c), *hæmleṭ* “Hamlet” appears either one or two clauses away from the embedded clause, but is still interpreted as the direct object of *poṛeche* “read.” The filler may appear either after the subject or before the subject, as in (15d).

(15)   a. jɔn    bhablo   [_S′_ meri bollo [_S′_ su hæmleṭ poṛeche read ]]
              John thought    Mary said    Sue Hamlet read
          b. jɔn    bhablo   [_S′_ meri **hæmleṭ** bollo [_S′_ su ____
               John thought   Mary Hamlet said    Sue
               poṛeche ]]
               read
          c. jɔn    **hæmleṭ** bhablo  [_S′_ meri bollo [_S′_ su ____
               John Hamlet thought    Mary said   Sue
               poṛeche ]]
               read
              ‘John  thought  that  Mary  said  that  Sue  has  read
               Hamlet’
          d. **hæmleṭ** jɔn    bhablo   [_S′_ meri bollo [_S′_ su ____
                Hamlet John thought    Mary said   Sue
                poṛeche ]]
                read
               ‘John  thought  that  Mary  said  that  Sue  has  read
                Hamlet’

Extraction from pre-verbal clauses is shown in (16). In (16a), the noun phrase *tomar beṛal-ke* “your cat-acc” is interpreted as the object of the embedded verb *kamṛeche* “bit,” but it appears in the left edge position of the main clause. Similarly, in (16b), the prepositional phrase *bas theke* “bus from” appears in the left edge position of the main clause, but is interpreted as a modifier of the embedded clause. This contrasts with other languages with both pre-verbal and post-verbal clauses, like Basque which disallows gap sites in pre-verbal clauses (Uriagereka, 1992), and Malayalam which only allows direct object gaps in pre-verbal clauses, but not for adjunct phrases like *bas theke* “bus from” (Srikumar, 2007). The filler may again either appear before the subject or after the subject, as in (16c).

(16)       a. **tomar beṛal-ke** amra šɔbai
                  your       cat-acc we     everyone
                  [_S′_ paš-er baṛi-r     kukur ___ kamṛeche bole ]
                      neighbor-gen dog         bit           that
                 šunechilam
                 heard
                ‘We had all heard that the neighbor’s dog has bitten
                  your cat'
              b. **bas theke** amar didi
                    bus from my     sister
                   [_S′_ ɔtogulo duronto       bacca laphiye
                     so many uncontrollable child jumping
                   nambe       bole ] bhabe ni
                  descend.fut that     think pst.neg
                  ‘My sister hasn’t thought that so many children could
                   jump down from a bus.
              c. amar didi     **bas theke**
                    my   sister bus     from
                  [_S′_ ɔtogulo duronto       bacca laphiye
                      so many uncontrollable child jumping
                  nambe       bole ] bhabe ni
                  descend.fut that     think pst.neg
                  ‘My sister hasn’t thought that so many children could
                   jump down from a bus.

To summarize, Bangla permits embedded clauses to precede or follow the embedding verb. Additionally, fillers in the main clause may resolve with gap sites in the main clause or in an embedded clause on either side of the embedding verb. This means the schematic representations in (17) are all permissible, making Bangla an excellent language for testing locality biases.

(17) a. Post-verbal embedded clause, main clause resolution:
               …***filler*** …___ …V …[_S′_…] …
          b. Post-verbal   embedded   clause,   embedded   clause   resolution:
                 …***filler*** …V …[_S′_…___ …] …
         c. Pre-verbal   embedded   clause,   embedded   clause   resolution:
               …***filler*** …[_S′_…___ …] …V …
         d. Pre-verbal embedded clause, main clause resolution:
               …***filler*** …[_S′_…] …___ …V …

If the locality constraints on preferred gap sites are sensitive to linear order, as suggested by findings in Japanese, then the dependencies schematized in (17a) and (17c) should be preferred to those in (17b) and (17d). However, if locality constraints on preferred gap sites are sensitive to structural locality, then the representations in (17a) and (17d) should be preferred, since the filler and gap site are structurally more local to the filler. We test these predictions in Experiments 1–3.

## Experiment 1

### Rationale

In Experiment 1, we used the Question after Story task (de Villiers et al., 1990) to determine whether Bangla speakers prefer linearly local gap sites across word orders. We adapted the design used by Omaki et al. (2013), which probed for word order effects on filler-gap dependency resolution using a between language comparison. In their study, participants viewed a series of vignettes in which a character acted out an event in one location and reported on it in another location. Afterwards, participants were asked to respond to a question that contained a fronted *wh*-filler that could resolve in either the embedded clause or main clause. Participants' responses revealed in which clause they preferred to resolve the filler-gap dependency. In English, a language that conflates linear and structural locality, the ambiguous filler-gap dependency was most commonly resolved with the main clause in Omaki and colleagues' studies. Conversely, in Japanese, the filler-gap dependency was preferentially resolved in the embedded clause. They took this as evidence for a universal preference to resolve filler-gap dependencies with the first position linearly available.

Our study took advantage of the flexible word order in Bangla to further test this hypothesis. The study had two main conditions: a main verb first condition, shown in (18a), and an embedded verb first condition, shown in (18b). For both sentences, the fronted *wh*-filler *kothae* “where” could be resolved in the embedded clause, modifying the catching event, or the main clause, modifying the telling event. If gaps are preferentially constructed in the first position linearly available, as suggested by Omaki and colleagues' cross-language contrast, then we expected *kothae* “where” to be resolved with the main verb in word orders like (18a), and with the embedded verb in word orders like (18b).

(18) a. **Main Verb First Condition**:
              šumi   kothae ækjɔn-ke     boleche [_S′_ je     še
              Shumi where someone-acc told     that she
              prɔjapoti dhorbe]?
              butterfly catch.fut
        b. **Embedded Verb First Condition**:
              šumi kothae [_S′_ še prɔjapoti dhorbe     bole]
              Shumi where     she butterfly catch.fut that
              ækjɔn-ke     boleche?
              someone-acc told
            “Where did Shumi tell someone that she will catch
              butterflies?”

### Participants

Ninety-six participants were recruited for Experiment 1. Forty-eight adult native speakers of Bangla were collected from the student population at The University of Dhaka in Dhaka, Bangladesh, and 48 participants were from the student population at Calcutta University in Kolkata, India. Bangladeshi participants were compensated 500 Bangladeshi Taka (BDT), and Indian participants were compensated 200 Indian Rupees (INR). This session took approximately 15 min. Experiment 1 was conducted after participants completed either Experiment 2 or after another experiment unrelated to the current study. These populations were each split into two groups, a “within-subjects” and a “between-subjects” group, as discussed in section Materials. We tested participants in both India and Bangladesh to probe for any potential influence of dialect difference, especially given that Indian Bangla speakers are likely to be competent in Hindi, which uses different *wh*-scope marking strategies (e.g., Dayal, 1996; Manetta, 2012). Additionally, we included a within-subjects and between-subjects manipulation to check for any effect of self-priming in the experiment. This was important for comparing our within-language findings to results from previous between-language comparisons, where participants in each language, e.g., Japanese and English, saw only one of the word orders tested in Bangla.

### Materials

The materials were adapted from Omaki et al. (2013). The stories and audio were translated by three of the authors to standard colloquial Dhakaiya Bangla. Some lexical material was changed to better suit the different cultural context, including names. The questions were presented on a paper questionnaire. Participants were instructed to respond to a question printed on the questionnaire immediately after each vignette, before progressing onto the next vignette. Across all questionnaires, we rigidly alternated between a target item and a filler item, in order to reduce priming or perseveration effects. The target items were two-clause sentences with an ambiguous *wh*-dependency, presented in (18). The fillers were one-clause sentences with an unambiguous *kæno* “why” question.

Participants were split into two groups—the “between participants” group and the “within participants” group. The “between participants” group was included to make a closer comparison to the existing literature comparing English and Japanese. The division of participants is illustrated in Table 1. Questionnaires were prepared for each group. For the “between participants” questionnaires, the target items all had either main verb first word orders or embedded verb first word orders, i.e., participants saw 4 target items in one of the two conditions. The remaining participants received a “within participants” questionnaire, where the target items contained both verb first word order and embedded verb first word orders, i.e., 2 target items per condition. In the within participants questionnaire, the two conditions alternated, such that there were two questions of each word order in each questionnaire.

**Table 1:** **Distribution of participants in experiment 1**.

**Total: 96**					
**Dhaka: 48**			**Kolkata: 48**		
**Within participants: 24**	**Between participants: 24**		**Within participants: 24**	**Between participants: 24**	
	Main verb first: 12	Embedded verb first: 12		Main verb first: 12	Embedded verb first: 12

*There were 96 participants in Experiment 1, 48 for each city, Dhaka and Kolkata. Each city was split into two groups. One group of 24 in each city saw both conditions in the same questionnaire, the within-participants group. Another group of 24 in each city was further divided in two groups of 12, one seeing only lists with main verb first word orders and the other seeing only lists with embedded verb first word order*.

The stories were animated vignettes made from clipart images. In each vignette, a character went to four different locations, and performed an action in each. A sample story from the English study in Omaki et al. (2013) is presented in (19). The videos are included as Supplementary Material.

(19) Sample story:
  [**Introduction**]
    It was a beautiful day in spring so Lizzie decided she was going to go catch butterflies in the park.
    [**1st Location**]
    Her Mom and Dad weren't home, so Lizzie thought she should tell her brother or sister about going to the park, so that Mom and Dad would know where she was when they got back. She first went to her brother's room, but he was taking a nap and she couldn't tell him about catching butterflies.
    [**2nd Location**]
    Instead, Lizzie looked for her sister. She looked all over the house but didn't see her sister anywhere! When she was about to give up, Lizzie heard her sister's voice in the basement! She went to the basement and said to her sister: “I'm gonna catch butterflies in the park!”
    [**3rd Location**]
    Then, on her way to the park, Lizzie passed by a parking lot and saw a butterfly near it. She walked slowly toward the butterfly, but before Lizzie could get there, another girl came along and caught the butterfly! Lizzie didn't see any more butterflies there, so she kept walking toward the park.
    [**4th Location**]
    There were lots and lots of butterflies in the park, and she caught one in a jar and took it home with her. She liked the one that she caught, but she wished she could have caught more butterflies.

**Figure 1:**
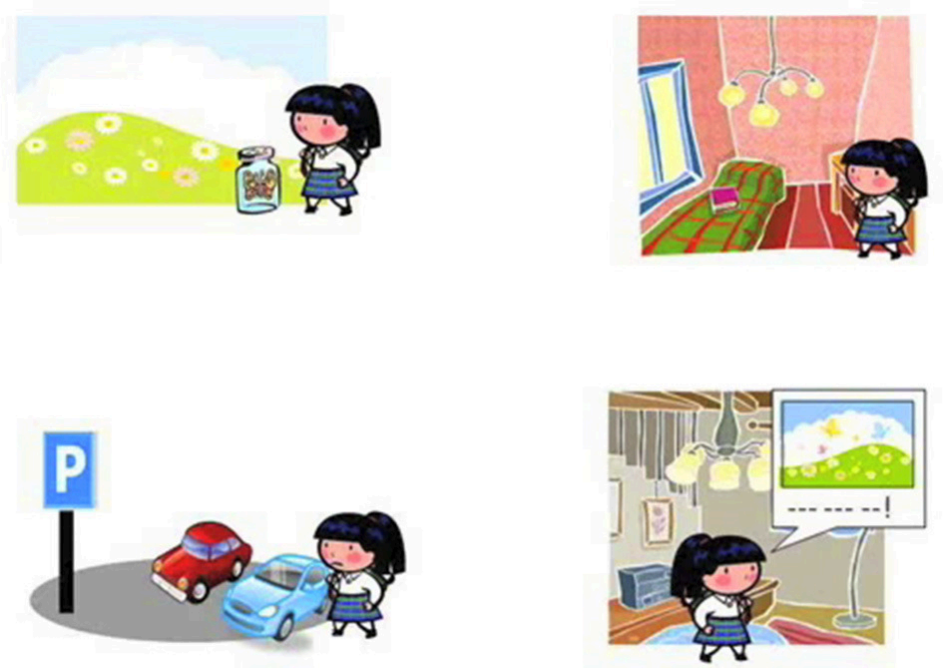
**Sample image from Experiment 1 materials**. In this vignette, the character Shumi successfully caught butterflies in the park, and reported on it in the first floor. The parking lot and bedroom are distractor locations.

Each vignette consisted of six phases. The first phase introduced the protagonist, displayed in the center of the screen. The following four phases depicted him or her at each of the four locations. The protagonist succeeded or failed to perform some intended action as announced in the introductory phase, or succeeded or failed to report on it. The contrast between successes and failures was intended to make the event-location pairings more memorable, and to ensure that the “where” test questions were felicitous. In locations where the protagonist succeeded on performing his or her stated action or reported on it, there was a visual trace left behind (i.e., a butterfly in a bottle, or a word balloon). The first two and last two locations were relevant for either the main clause event (i.e., the reporting event), or the embedded clause event (i.e., the intended action). In the sixth and final phase, the protagonist returned to the center of the screen, and then the story concluded. A sample image from the vignette is given in Figure 1.

To avoid any potential recency bias, the ordering of the events within each story was counterbalanced, such that the first pair of events pertained to the reporting event in half of the stories, and to the embedded clause event in the other half of the stories. In each case, the story provided motivation for continuing to the next series of events. For instance, In (19), the reporting events are motivated by the character's need to tell her siblings where she was going. The pairings of quadrant position and event were randomized across stories so that participants could not predict which locations would correspond to which actions.

### Methods

Experiment 1 was an adaptation of Omaki et al. (2013), question-after-story task (de Villiers et al., 1990). Participants were instructed in Bangla to watch a sequence of 8 vignettes. At the end of each vignette, the screen displayed “write your answer now” in Bangla. At this point, the experimenter paused the video and instructed the participant to read a question printed on a paper questionnaire. Participants were instructed to write a brief response. We asked that the responses be brief because in pilot studies, participants attempted to recapitulate large portions of the story, which complicated coding the results. After responding, the experimenter resumed the video, which progressed to the next vignette.

### Results

We coded each response as either a main clause response or an embedded clause response, depending on which location the participant named. Responses that either failed to answer the question or that provided both possible answers were excluded. Most of the excluded responses named both possible locations, implying that Bangla speakers were often aware of the ambiguity. The proportions of excluded observations are given in Table 2.

**Table 2:** **Proportion of removed responses in Experiment 1**.

	**Dhaka**		**Kolkata**	
	**Between participants (%)**	**Within participants (%)**	**Between participants (%)**	**Within participants (%)**
Main verb first	25	29	31	21
Embedded verb first	15	33	8	23

There were fewer exclusions for the embedded verb first conditions in the between-participants conditions compared to other conditions. This is the only list in which participants saw only the canonical, verb-final word order. This is because the fillers across all lists used this word order, and all target items in this list also used embedded verb first word order. The presence of non-canonical word orders in other lists may have made the ambiguity more salient, leading to a higher number of exclusions. After excluding these observations, participants responded with the main verb location in 81% of the main verb first word orders, but only 23% of the embedded verb first word orders.

**Figure 2:**
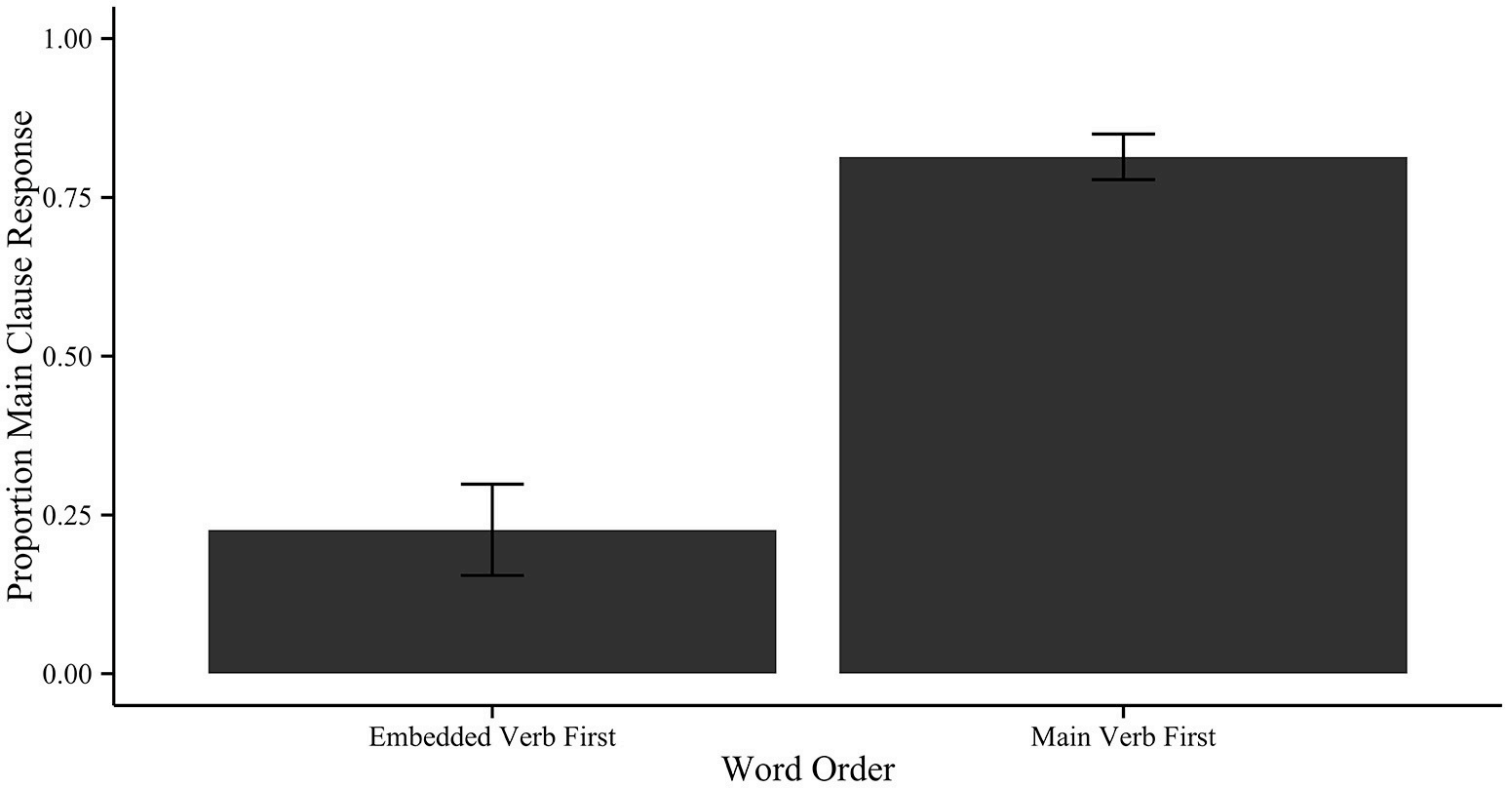
**Proportion main clause response by word order in Experiment 1**. Error bars correspond to one standard deviation from the mean. Proportions are collapsed across list types and locations.

Using the lmer package in R (Bates et al., 2015), we submitted the results to a logit mixed effects model with a *bobyqa* optimizer. The predicted variable was main clause response, coded as 1. For fixed effects, we included word order (main verb first or embedded verb first), location (Dhaka or Kolkata), and list type (within participants or between participants), with their interaction terms. We included these factors in order to fit a maximal model that tested for all potential variables of interest. For random effects, we included participant and items. Afterwards, we used the backward elimination method to eliminate factors from the model one-by-one to minimize the AIC (Akaike Information Criterion) of the model, as described by Faraway (2002). The results of the best-fit model are given in Table 3. The *p*-values in Table 3 were generated using the lmerTest package (Kuznetsova et al., 2015). The mean proportion of main verb responses is actually given in Figure 2.

**Table 3:** **Results of best-fit logistic regression model for Experiment 1**.

**Fixed effects**	**Estimate**	***SE***	***z***	***p***
(Intercept)	−2.03	0.97	−2.08	0.04^*^
Word order	5.16	0.95	5.41	<0.001^*^
City	−1.12	0.92	−1.22	0.22
List type	−0.96	0.91	−1.05	0.29
City ^*^ list type	2.62	1.34	1.96	0.05

*P-values lower than 0.05 are marked with an asterisk*.

We found a significant effect of word order on the proportion of main clause responses. The effect was as predicted: for the main verb first word order, participants showed a strong bias to answer with main verb locations. With embedded verb first word orders, there was a strong bias to answer with embedded verb locations. There was no significant effect of city, implying that there were no systematic dialect differences detected in Experiment 1. Additionally, there was no significant effect of list type, i.e., participants typically responded with the event denoted by the first verb linearly available regardless of whether they saw lists with only one word order or lists with mixed word order. However, there was a marginal interaction of city and list type, due to an increase in main clause responses for the Kolkata participants in the within-participants list (β = 2.61, *SE* = 1.33, *z* = 1.96, *p* = 0.0504). This suggests that participants from Kolkata may have a main clause preference when exposed to both word orders, although the effect of interest persists even in this population.

For the main verb first word order, participants responded with the location denoted by the main verb in 72% of the trials in the within-participants list, and 81% of the trials in the between-participants list. For the embedded verb first word order, participants responded with the location denoted by the main verb in 28% of the within-participants trials, and 19% of the between-participants trials. Thus, we replicated Omaki and colleagues' cross-language findings in the between participant group, and showed a robust bias to resolve the filler-gap dependency with the first verb across word orders in the within participant group as well.

### Discussion

In Experiment 1, we showed that Bangla speakers preferentially resolved a filler-gap dependency with the first position linearly available, regardless of whether this position was in the same clause as the filler or in a more deeply embedded clause. This suggests that the locality constraints determining preferred gap sites are primarily sensitive to linear distance, as previously shown in a between-language comparison by Omaki et al. (2013). Importantly, this contrasts with observations about island constraints, which appear to be defined in terms of hierarchical structure.

This within-language demonstration of sensitivity to linear order is also important because it helps keep constant all other grammatical properties between the word order comparisons. The results found by Omaki and colleagues may be due to some other grammatical distinction between English and Japanese apart from word order. For instance, obligatory long-distance *wh*-dependencies as observed in English have different properties than the optional *wh*-dependencies observed in Japanese (“scrambling,” Saito, 1985; Mahajan, 1990), which might indirectly bias the filler-gap dependency resolution preferences in these languages. These concerns are less likely to impact the results of Experiment 1, particularly because the effect is robust in the within participant questionnaires. We cannot exclude the possibility that there are subtle formal differences between the pre-verbal and post-verbal filler-gap dependencies. But even if there are such differences, extant accounts of filler-gap dependency processing do not predict that such fine-grained differences should have a large effect on locality biases. We therefore take our findings to lend support to the notion of a general linear locality bias in filler-gap dependency processing.

One potential concern is that the sentences in the embedded-verb first condition may have been parsed as unambiguous. Since the question word *kothae* “where” in (18b) appeared adjacent to the embedded subject it may have been parsed as having a surface position inside the embedded clause. That is, the filler may have been entirely contained in the embedded clause, requiring an embedded clause interpretation. If so, then the embedded clause responses clearly would have been required. However, we consider this unlikely, since these conditions elicited 23% main verb responses, plus additional (excluded) responses in which participants mentioned both possible answers. So, we think that it is unlikely that these sentences were surface unambiguous for our participants.

An advantage of the Question after Story task in Experiment 1 is that it directly probed participants' preferred resolution sites instead of measuring measuring whether they detect an unexpected parse, as in the filled-gap effect. However, the Question after Story task does not reveal the time course of dependency formation. We cannot infer from these data that there is early commitment to the linearly first gap site. For this reason, in Experiment 2, we used a filled-gap paradigm in a self-paced reading task to probe for detection of an unsubstantiated gap expectation across word orders.

## Experiment 2

### Rationale

In Experiment 1, we showed that comprehenders preferred to resolve filler-gap dependencies with the first verb linearly available. The goal of Experiment 2 was to test whether this follows from an early and confident commitment to this gap location. We used the filled-gap paradigm in a self-paced reading task (Crain and Fodor, 1985; Stowe, 1986), which tests whether participants can immediately detect that a previously constructed gap is unavailable. If commitment to the first gap site is made early and confidently enough across word orders, then we expected a filled-gap effect when filler-gap dependency resolution with the first verb was blocked, regardless of whether this occurred in the same clause as the filler or in an embedded clause.

### Participants

Participants were 32 adult native speakers of Bangla from the University of Dhaka student community. Due to a technical error, 3 participants' responses were not recorded, and thus we report on 29 participants. They were compensated 500 BDT for their time. The task took approximately 20–30 min to complete.

### Materials

We crossed the factors word order (main verb first or embedded verb first) and extraction type (argument or adjunct extraction). In all target items there was a long-distance *wh*-filler gap dependency. The critical conditions contained an argument *wh*-filler (*ka-ke* “who-acc”) marked in the accusative case. This argument *wh*-filler was blocked from resolving with the linearly first gap position by a case-matching noun phrase occupying the first canonical object position. This was the filled-gap region and the critical region. The adjunct extraction conditions (*kkhon* “when,” *kothae* “where”) were the control conditions, since the accusative-marked noun phrase did not block resolution of an adjunct *wh*-dependency in that clause. Table 4 gives a sample set of items, with the critical filled-gap region underscored and the regions delimited by pipes. Regions were predominantly one word each, except for certain compound verb constructions which contained two words but were treated as one region in the analysis. There were 15 regions in each word order condition, with the filled-gap region being the 7th region in the main-verb first word order, and the 8th region in the embedded-verb first word order.

**Table 4:** **Sample materials from Experiment 2**.

***rašad |***	***jiggæša |***	***koreche |***	***{ka-ke|kothae} |*** …		**{Argument|Adjunct}**						
**Rashad**	**ask**	**did**	{**who|where**} …								
**Main Verb First:**			…*ḍakṭar-ṭa* |	*khubi* |	*bakbhabe* |	*rugi-ke* |
			…doctor-cl	very	surprisedly	patient-acc
			*bolechen* |	*je* |	*tini* |	*purano*
			told	that	he.pol	old
			*haspatal-e* |	(*take*) *|*	*cikitša* |	*korechen*					
			hospital-loc	(him)	treatment	did					
“…the doctor told the patient that he treated (him) in the old hospital”											
**Embedded Verb First:**			…*ḍakṭar-ṭa* |	*tini* |	*purano* |	*haspatal-e* |
			…doctor-cl	he.pol	old	hospital-loc
			*rugi-ke* |	*cikitša* |	*korechen* |	
			patient-acc	treatment	did	
			*bole* |	*khubi* |	*bakbhabe* |	(*take*) | *bolechen*
			that	very	surprisedley	(him) told
“…the doctor told (him) that he treated the patient in the old hospital”											

*Critical filled-gap region is underlined, and regions are demarcated by pipes*.

In the main verb first conditions, the argument *wh*-filler was blocked from resolving as the indirect object of the verb *boleche* “said/told.” The *wh*-filler must then resolve as the direct object of the later, embedded verb. Conversely, in the embedded verb first conditions, the argument *wh*-filler was blocked from resolving as the direct object of the embedded verb. It must therefore resolve as the indirect object of the main verb *boleche*. In the adjunct extraction conditions, an extra pronoun *take* “him/her-acc” was introduced as the object of the embedded verb in main verb first conditions, and the verb *boleche* “said” in the embedded verb first conditions. This was necessary to ensure that all verbs had all argument roles discharged. In all conditions, the fronted *wh*-phrase appeared on the left edge of its containing clause to maximize the distance between the *wh*-phrase and the filled-gap region. This prevented the filler from being analyzed as left-adjoined to the embedded clause in the embedded clause first condition. The adjunct *wh*-phrases were counterbalanced between *kkhon* “when” and *kothae* “where.” The subject of the main clause containing the *wh*-filler always denoted a referent of high status, and the pronoun in the most deeply embedded clause and its verb were morphologically marked with politeness agreement (*tini*). This was done to minimize the complexity induced by any retrieval operations needed in each pronoun and verb region, by maximizing the distinguishability of the referents introduced in the sentence. Additionally, this prevented a potential misanalysis, since a demonstrative is sometimes spelled homographically with the informal third person pronoun (š*e*). All target conditions were embedded in an additional clause (*rašad jiggæša koreche…* “Rashad asked…”). This was to ensure that participants could not predict the word order of the target items on the basis of the first few words. There were 32 sets of target items and 48 complexity-matched fillers. The sentences were presented in a Latin Square design, with order randomized for each participant.

### Methods

Sentences were presented on a PC laptop using the Ibex software (http://www.spellout.net/ibexfarm) in a self-paced, word-by-word, moving window paradigm (Just et al., 1982). Ibex is intended for web-based tasks, but the task was run offline by one of the authors. Each trial began with a screen presenting a sentence in which the words were masked by dashes, with spaces intact. Each time the participant pressed the spacebar, a word was revealed and the previous word was again hidden behind a dash. A yes/no comprehension question appeared all at once after the participant completed each sentence. The participant was instructed to use the “f” key to respond “yes,” and the “j” key to respond “no,” with on-screen reminders of this key-response pairing. On-screen feedback informed the participant whether the response was correct. Participants were instructed to read carefully at a natural but quick pace, and to answer the questions carefully. The order of presentation of responses was randomized for each participant. All instructions and feedback were given in Bangla.

### Results

Analyses were conducted on comprehension task accuracy and reading times. Trials that received incorrect responses in the comprehension task were removed from analysis. Four participants whose mean accuracy fell below 70% were removed from analysis. The mean accuracy on the comprehension questions was 80.6% after removing these 4 participants.

**Figure 3:**
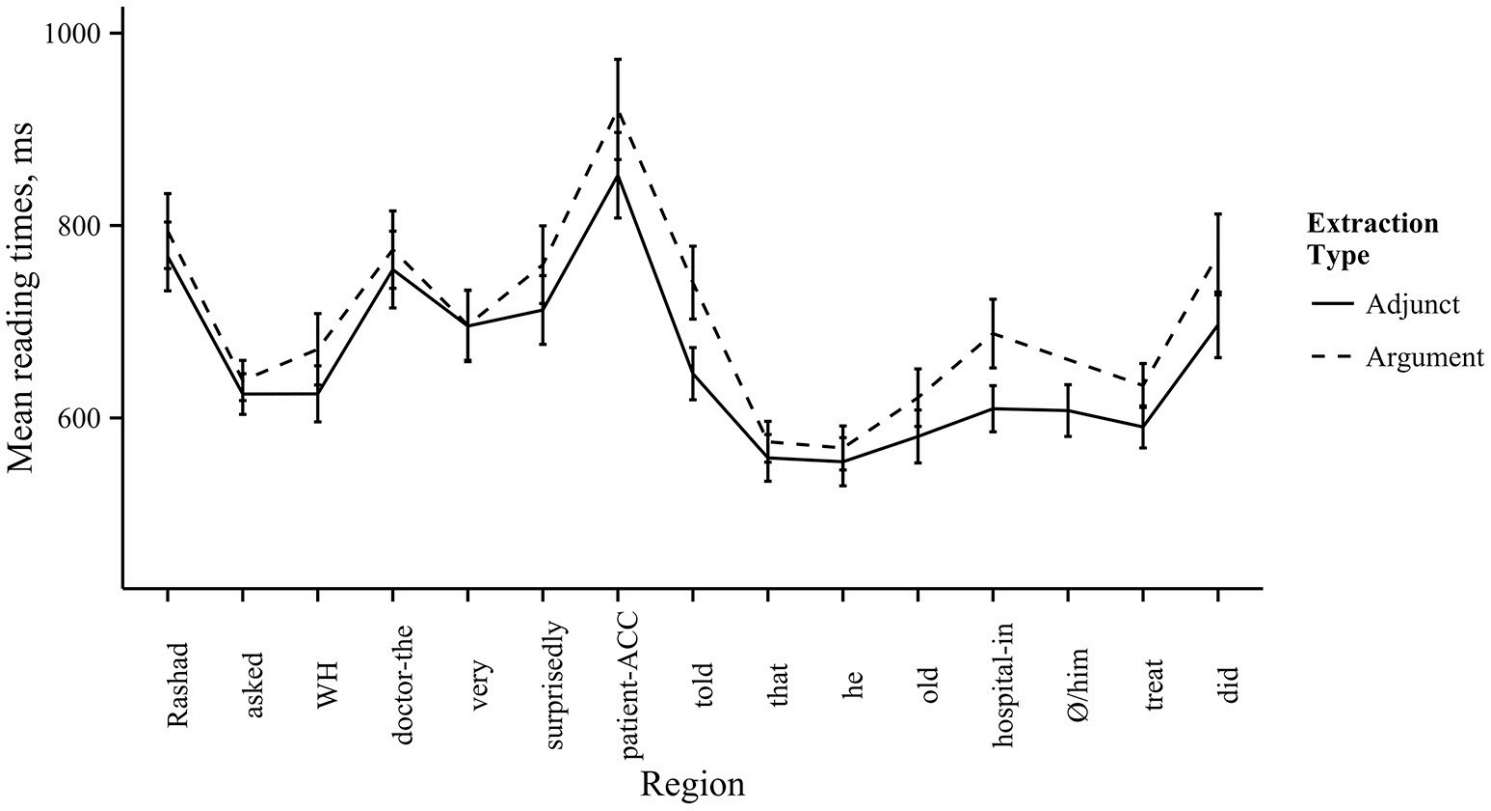
**Reading times by region in Experiment 2, main verb first conditions**. Mean reading times by region. Line type corresponds to extraction type. Error bars represent one standard error from the mean.

**Figure 4:**
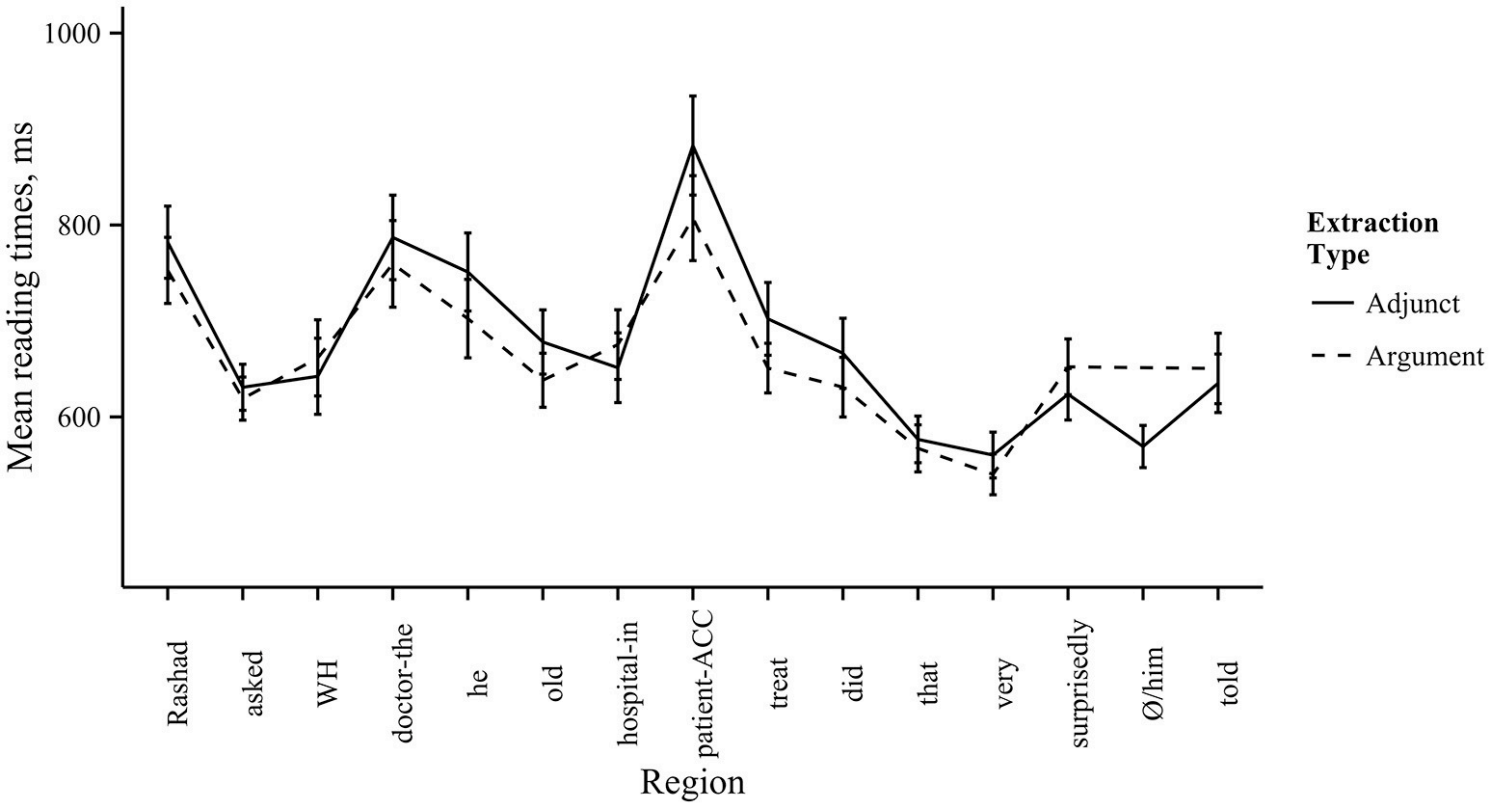
**Reading times by region in Experiment 2, embedded verb first conditions**. Mean reading times by region. Line type corresponds to extraction type. Error bars represent one standard error from the mean.

Using the lme4 package in R (Bates et al., 2015), we analyzed the reading times for the filled-gap region and the subsequent regions using linear mixed effects models for each word order. We included log-transformed reading times as the predicted variable, and extraction type (argument vs. adjunct) as the predictor factor. We also included random intercepts for participants and items. For the main verb first conditions, we found no effect of extraction type in the filled-gap region [7th region, *rugi-ke* “patient-acc,” β = 0.04, *SE* = 0.04, *t*_(259)_ = 1.0, *p* = 0.32]. However, in the region immediately following the filled-gap region, there was a main effect of extraction type, due to longer reading times in the argument extraction condition [8th region, *boleche* “said,” β = 0.08, *SE* = 0.04, *t*_(270)_ = 2.1, *p* = 0.04]. This indicates a filled-gap effect for the main verb first word order, suggesting that readers made an early commitment to a gap for the *wh*-filler in this position. Additionally, we found a significant effect of extraction type in the embedded clause, due to longer reading times in the argument extraction condition [12th region, *haspatal-e* “hospital-in,” β = 66.41, *SE* = 28.87, *t*_(268)_ = 7.3, *p* = 0.02]. This may reflect a secondary filled-gap effect, since it occurs two regions after the embedded clause subject. Comprehenders may have attempted to resolve the filler-gap dependency with the embedded clause subject position. However, this would imply that Bangla speakers do not use case information to determine resolution sites, contrary to our assumptions. Thus, we do not have a good explanation for why reading times should increase at this region. For the embedded verb first conditions, there was no effect of extraction type at the filled-gap region [8th region, *rugi-ke* “patient-acc,” β = −0.03, *SE* = 0.04, *t*_(285)_ = −0.69, *p* = 0.49] or in the following two regions [9th region, *cikitša* “treatment,” β = 0.05, *SE* = 0.04, *t*_(302)_ = 1.3, *p* = 0.20; 10th region, *korechen* “did,” β = 0.003, *SE* = 0.004, *t*_(300)_ = 0.08, *p* = 0.94]. Thus, we found no filled-gap effect in the embedded verb first word order. The mean reading times by region are given in Figures 3, 4.

### Discussion

Experiment 2 was designed to probe for sensitivity to blocking of a preferred gap location across word orders using an on-line measure. If the preference for linearly local gaps found in Experiment 1 reflects an early and confident commitment in both word orders, then we predicted sensitivity to disruption when this resolution was unavailable in the filled-gap paradigm. However, we only found sensitivity to disruption in the main verb first word orders, i.e., when structural locality and linear locality converged. In other words, we did not find evidence of early commitment to this position in Experiment 2. This suggests that the class of gaps that are preferred is not identical to the class of gaps that are committed to early enough to elicit a filled-gap effect.

This difference in measures may be due to a selective sensitivity to structural locality. For instance, the bias to resolve with the first gap linearly available may only manifest as an early commitment when this position is also structurally local. If the biases for preferred gap sites are sensitive to structure in this way, then this undermines one argument for the separation of island constraints from biases on preferred gap sites, i.e., the argument that they should be separated because they refer to different properties of the representation.

However, the differences between the results in Experiments 1 and 2 may reflect differences between the tasks. Experiment 1 probed directly for resolution preferences in an off-line task. The sentences were globally ambiguous, and somewhat simpler than the three-clause sentences in Experiment 2. Conversely, Experiment 2 was an on-line reading task in which participants read sentences word-by-word.

Participants also seemed to have some difficulty with this task, since their accuracy on the comprehension questions are somewhat lower than average. Additionally, in pilot versions of Experiment 2, participants read at very different paces. Furthermore, participants reported different levels of familiarity with computers, which may have exacerbated the unnaturalness of the task. Some participants struggled with the instructions, e.g., some participants held the space bar down, failing to release it between words. These additional complications in Experiment 2 may have masked an early commitment to resolve a filler-gap dependency with the first gap linearly available, even in embedded verb first word orders. In other words, participants may have made an early commitment to resolve with a linearly local gap site in embedded verb first word orders, but this was selectively masked in Experiment 2.

In Experiment 3, we address these cross-experimental concerns by using the filled-gap paradigm in an offline acceptability judgment task. Experiment 3 was an off-line acceptability judgment task that used two-clause sentences. This was intended to make it as similar to Experiment 1 as possible. The target sentences all contained a filled-gap in a linearly local position, as in Experiment 2. Thus, Experiment 3 relied on an indirect measure of disruption like Experiment 2. Additionally, Experiment 3 was an untimed pen-and-paper task, like Experiment 1, removing the on-line aspect of the self-paced reading paradigm in Experiment 2. If we find evidence of sensitivity to disruption only in main verb first word orders in Experiment 3, i.e. when structural and linear locality converge, then we can conclude that structural locality affects the processes involved in making an early commitment to a gap site. Conversely, if there is a filled-gap effect in Experiment 3 across word orders, then we can infer that the failure to find a filled-gap effect in Experiment 2 was due to the design of that experiment.

## Experiment 3

### Rationale

In Experiment 3, we again investigated whether Bangla speakers preferred gap sites that are linearly local or structurally local. In Experiment 1, we found evidence for an off-line bias for linearly local gap sites. In Experiment 2, we found sensitivity to disruption with a filled-gap in a linearly local position, but only with main verb first word order, i.e., when the first gap was also structurally local. In Experiment 3, we investigated whether this mismatch between the results in Experiments 1 and 2 was due to the on-line/off-line contrast between the studies, or the ambiguity resolution/filled-gap paradigm contrast.

Experiment 3 was an off-line acceptability judgment task. In this task, participants read sentences in an untimed way, as in Experiment 1. However, like in Experiment 2, we used a filled-gap paradigm. Although the filled-gap paradigm is typically used in on-line measures, it can also be used to detect filled-gap effects in off-line measures (Sprouse, 2008). This is because the reanalysis associated with detecting a filled-gap effect also lowers ratings in acceptability judgment tasks. Thus, we can compare the ratings for sentences in which the preferred gap is unavailable with controls. If we find a decrease in acceptability, then we take this to be a filled-gap effect. If we find a filled-gap effect across both word orders in Experiment 3, then we can infer that the lack of an embedded clause bias in embedded verb first word orders is due to the design of Experiment 2. Conversely, if we find evidence for a filled-gap effect in the main verb first word order only, then this implies that the difference between Experiments 1 and 2 may be due to the different nature of ambiguity resolution tasks (Experiment 1) and the mechanisms involved in detecting filled-gap effects (Experiments 2–3).

### Participants

Participants were adult native speakers of Bangla drawn from the University of Dhaka and Calcutta University student populations. There were 32 participants from each group. Participants in Dhaka were compensated 500 BDT for their time, and participants in Kolkata were compensated 200 INR. The experiment lasted approximately 10–20 min, and was conducted after either Experiment 2 or another unrelated experiment.

### Materials

The materials in Experiment 3 were constructed in a similar way to the materials from Experiment 2. We crossed three factors—word order (main verb first or embedded verb first), extraction type (argument extraction or adjunct extraction), and filled-gap position (linearly local or linearly distant). This third factor was added to test for any filled-gap effect with the main clause verb in embedded verb first word orders, i.e., to probe for a filled-gap effect in a position that was linearly distant but structurally local. We constructed 8 lists with an equal number of items per condition, and an equal number of items across lists. There were 24 sets of target items, and 36 complexity-matched fillers, 18 of which were ungrammatical. Each participant saw 3 sentences from each condition and all the fillers in a randomized order.

There were a few differences between the target items in Experiments 2 and 3 that are worth noting. First, the target items in Experiment 3 contained two clauses, unlike the three clause sentences in Experiment 2. This is more similar to the materials in Experiment 1. Additionally, the *wh*-phrase appeared in the pre-verbal position like in Experiment 1, not the left-edge position as in Experiment 2. This was done because the pre-verbal position is perhaps the more canonical position for *wh*-fillers (Simpson and Bhattacharya, 2003). Since the *wh*-filler is in its canonical position adjacent to the embedded clause in embedded verb first word orders, it is possible that comprehenders will treat this as unamibiguous, as we suggested in “Section Discussion”. This should bias the results to have an embedded resolution with embedded verb first conditions. Lastly, in the adjunct conditions we did not include the additional object pronoun. In Experiment 2, we included this extra pronoun to ensure that the verb with which the argument *wh*-filler was interpreted had an overt argument in the adjunct extraction conditions. However, this may have been unnecessary, since Bangla permits null arguments. Thus, we did not include this extra pronoun, to maximize similarity between the argument and adjunct extractions. A sample set of materials is given in Table 5.

**Table 5:** **Sample materials for Experiment 3**.

*Jahid* *{**ka-ke|kothae**}* …	**{Argument|Adjunct}**
Jahid {who-acc|where}…	
**Main V, Local FG**	…*khubi ɔbakbhabe tar bondhu-ke boleche je nipa parṭy-te dekheche*
	…very surprisedly his friend-acc said that nipa party-at saw
	“Who/where did Jahid very surprisedly tell his friend that Nipa saw at the party?”
**Main V, Distant FG**	…*khubi ɔbakbhabe boleche je nipa parṭy-te tar bondhu-ke dekheche*
	…very surprisedly told that nipa party-at his friend-acc saw
	“Who/where did Jahid very surprisedly tell that Nipa saw his friend at the party?”
**Embedded V, Local FG**	…*nipa parṭy-te tar bondhu-ke dekheche bole khubi ɔbakbhabe boleche*
	…Nipa party-at his friend-acc saw that very surprisedly told
	“Who/where did Jahid tell very surprisedly that Nipa saw his friend at the party?”
**Embedded V, Distant FG**	…*nipa parṭy-te dekheche bole khubi ɔbakbhabe tar bondhu-ke boleche*
	…Nipa party-at saw that very surprisedly his friend-acc told
	“Who/where did Jahid tell his friend very surprisedly that Nipa saw at the party?”

### Methods

Experiment 3 was a pen-and-paper acceptability judgment study. Participants were instructed to read the sentences carefully, and then circle a number ranging from 1 to 7, with lower scores indicating unacceptability. They were given example sentences with values already circled to illustrate how to use the scale.

### Results

**Figure 5:**
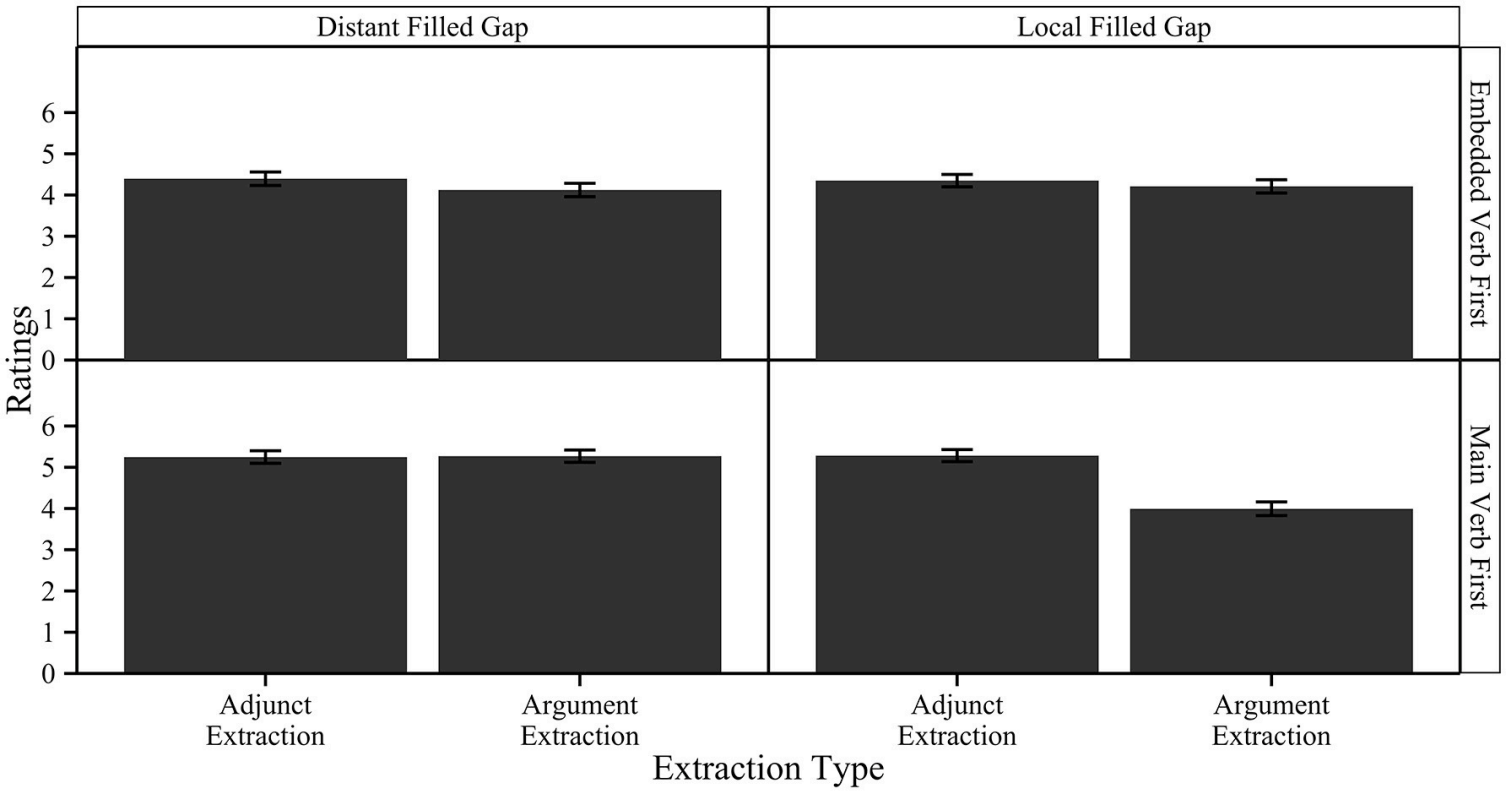
**Ratings by condition in Experiment 3**. Error bars represent standard errors of the mean.

We submitted the ratings to a linear mixed effects model, using the lme4 package in R (Bates et al., 2015). We included random effects for participant and item. We included word order (main verb first or embedded verb first), extraction type (argument extraction or adjunct extraction), and filled-gap position (linearly local or linearly distant) as predictors, together with their interaction terms. We also included location (Dhaka or Kolkata) in the model. We then used the backwards elimination method to simplify the model using the step() function in R, eliminating the location factor. The estimates of the model are presented in Table 6. The means of the ratings by condition are given in Figure 5. We then performed pairwise comparisons for extraction type within the two word orders and two filled-gap positions, using the least-squares means estimates with Tukey adjustment. These are shown in Table 7.

**Table 6:** **Results of best-fit mixed effects model for Experiment 3**.

**Fixed Effects**	**Estimate**	***SE***	***t***	***df***	***p***
(Intercept)	4.39	0.18	24.2	281	<0.0001^*^
Word order	0.87	0.20	4.25	1451	<0.0001^*^
Filled-gap position	−0.04	0.20	−0.21	1451	0.83
Extraction type	−0.25	0.20	−1.25	1451	0.21
Word order ^*^ FGPosition	0.06	0.29	0.21	1452	0.84
Word order ^*^ ExtType	0.24	0.29	0.85	1452	0.40
FGPosition ^*^ ExtType	0.11	0.28	0.39	51	0.70
WOrder ^*^ FGPosition ^*^ ExtType	−1.39	0.40	−3.43	1451	0.0006^*^

*P-values lower than 0.05 are marked with an asterisk*.

**Table 7:** **Results from pairwise comparisons in Experiment 3**.

**Word order, filled gap position**	**Estimate**	***SE***	**t ratio**	***df***	***p***
Main verb first, local filled gap	1.28	0.20	6.38	1450	<0.0001^*^
Main verb first, distant filled gap	0.009	0.20	0.043	1436	0.97
Embedded verb first, local filled gap	0.14	0.20	0.70	1428	0.48
Embedded verb first, distant filled gap	0.25	0.20	1.24	1433	0.22

*Comparisons were between argument and adjunct extractions within word order and filled-gap position. Comparisons were made using least squares means with Tukey HSD adjustment. P-values lower than 0.05 are marked with an asterisk*.

There were two main findings in Experiment 3. First, we found a main effect of word order. Ratings were significantly increased in main verb first word order [β = 0.87, *SE* = 0.20, *t*_(1451)_ = 4.25, *p* < 0.001]. This is consistent with the observation that main verb first word orders are the preferred word order for clausal embedding in Bangla. Secondly, there was a three-way interaction between word order, filled gap position, and extraction type [β = −1.39, *SE* = 0.40, *t*_(1451)_ = −3.4, *p* < 0.001]. The only significant pairwise comparison was between argument and adjunct extraction in the main verb first, local filled gap conditions [β = 1.28, *SE* = 0.20, *t*_(1450)_ = 6.38, *p* < 0.0001]. This reflects the lowered ratings with main verb first word order, local filled gap, and argument extraction conditions. In other words, there was a decrease in ratings when an argument *wh*-filler could not resolve with the main verb in main verb first word orders. This is a replication of the filled-gap effect in Experiment 2 in off-line acceptability judgments. Crucially, this was only observed in the word orders in which linear and structural locality aligned, i.e., we only found a filled gap effect in situations where the first potential gap position was both structurally and linearly local. Additionally, there was again no difference between participants in Dhaka or Kolkata, suggesting that there is no difference between dialects.

### Discussion

The goal of Experiment 3 was to determine whether Bangla speakers are sensitive to disruption of a linearly local filler-gap dependency resolution using an off-line measure. We conducted Experiment 3 to determine whether the lack of a filled-gap effect in embedded verb first word orders in Experiment 2 was due to the design of that experiment, or whether it reflects a difference between sensitivity to disruption and general locality preferences, as explored in Experiment 1.

The results from Experiment 3 show that Bangla speakers are only sensitive to disruption of a linearly local filler-gap dependency resolution in main verb first word orders. There was no filled-gap effect with linearly distant filled-gaps in either word order, and there was no filled-gap effect with linearly local filled-gaps in embedded verb first word orders. In other words, we again found a contrast between word orders with respect to sensitivity to disruption with the linearly local gap position.

Thus, we conclude that there is a general bias to resolve filler-gap dependencies with the first position linearly available, but that this only translates into an early and confident commitment when this is also a structurally local position. This means that the bias to resolve with the first position linearly available is only one component of detecting a disrupted filler-gap dependency, as measured with paradigms like the filled-gap effect.

## General discussion

In this paper, we investigated filler-gap dependency formation in Bangla. Bangla features flexible word order that permits us to manipulate whether the first position linearly available is in an embedded clause or in the main clause. This allows us to manipulate whether linear and structural locality diverge. In main verb first word orders, the first gap position linearly available is also structurally local, whereas in embedded verb first word orders the first gap position linearly available is structurally distant. In Experiment 1, we found a strong bias to resolve an ambiguous filler-gap dependency with the first position linearly available, regardless of its structural depth. However, in Experiments 2 and 3, we only found evidence of sensitivity to disruption when linear and structural locality converge. We interpreted these results as showing that there is a general bias for linearly local gaps, but that this only translates into a strong early commitment to this gap site when the linearly local gap is also structurally local.

If we start with the assumption that preferred gaps are typically detectable in a filled-gap paradigm, then the finding that these measures diverge for embedded verb first word orders in Bangla is surprising. The filled-gap paradigm, an indirect measure of gap formation preferences that we used in Experiments 2 and 3, depends on multiple processing mechanisms. The paradigm requires that participants make an early and confident commitment to a gap site, presumably in accordance with their linear locality preferences. Upon encountering the filled gap, the comprehender must quickly detect that the preferred gap is unavailable, and then instigate a costly reanalysis process. The lack of a filled gap effect in embedded verb first word orders might be attributed to any of these processes failing to deploy quickly.

Bangla speakers may not have shown a filled-gap effect in embedded verb first conditions in Experiments 2 and 3 because this word order is dispreferred. This was reflected in the lowered ratings for this word order in Experiment 3. This may be in part because pre-verbal embedded clauses have specific semantic and syntactic restrictions, unlike post-verbal embedded clauses (e.g., Bayer, 2001). In our estimation, long pre-verbal embedded clauses are also likely less frequent in naturalistic speech, and may carry certain pragmatic or discourse functions that also make them atypical. As a consequence, Bangla speakers may find processing pre-verbal embedded clauses more difficult, and have less facility making fine-grained predictions in pre-verbal embedded clauses for that reason. This contrasts with Japanese, in which pre-verbal embedded clauses are canonical (Tanaka, 2001), and filled-gap effects are found in pre-verbal embedded clauses (Aoshima et al., 2004; Yoshida, 2006).

Another salient difference between Bangla and Japanese is the case system. In Japanese, nominative marking surfaces as a morpheme –*ga*, and is used to quickly detect embedded clauses in real-time processing (Miyamoto, 2002). However, Bangla nominative noun phrases are morphologically unmarked. We speculated in Section Grammatical Properties of Bangla that Bangla speakers should be able to detect an embedded clause in embedded verb first word orders by observing two animate noun phrases, zero-marked for nominative. This is because case-marking morphemes are obligatory for animate noun phrases in non-subject positions. However, Bangla speakers might not compute this immediately. If Bangla speakers cannot immediately detect the embedded clause nominative subject as such, then construction of the embedded clause might be delayed, potentially even until the embedded verb. The relative timing of the construction of the embedded clause could explain the difference between these languages and the two measures in Bangla. Aoshima et al. (2004) suggested that the embedded clause bias in Japanese follows from a reanalysis triggered by the onset of the embedded clause. They propose that a Japanese comprehender first commits to a main clause gap position for a filler-gap dependency, and then revises to an embedded clause interpretation upon detecting the embedded clause. Crucially, this means that the comprehender has committed to a gap site in the embedded clause before encountering the filled-gap. If Bangla speakers cannot detect the embedded clause until after the filled gap, then there is no commitment to a gap resolution in the embedded object position by the time the comprehender enters that region. Thus, Bangla speakers should not show any evidence of reanalysis in the filled gap region. On this view, we predict no filled-gap effect in this context (as observed in Experiment 2), nor any reduction in judgments associated with such a reanalysis (as observed in Experiment 3). However, by hypothesis, Bangla speakers still have an embedded clause preference, and in off-line tasks they eventually select an analysis where a filler-gap dependency resolves in this position, if the string permits it. Thus, measures that directly probe for preferences in ambiguity resolution are predicted to reveal an embedded clause resolution preference when this position is linearly first, as in Experiment 1. A clear prediction of this account is that a language that has word order flexibility like Bangla but a case marking system like in Japanese should exhibit filled-gap effects in pre-verbal embedded contexts. An explanation that leverages these differences in information flow due to differences in case-marking may be the most promising framework for explaining these apparent differences.

Another possibility is that the contrast between our experiments is due to differences between argument and adjunct *wh*-dependencies. Experiment 1 tested adjunct *wh*-questions, whereas Experiments 2 and 3 tested argument *wh*-questions for the target conditions. This was by design, because adjuncts more easily permit the crucial ambiguity in Experiment 1, and argument *wh*-questions are more amenable to the filled-gap paradigm. However, there is little existing evidence that argument and adjunct *wh*-dependencies are comprehended differently. If resolving filler-gap dependencies is motivated by the need to find a semantic role for the unintegrated filler, then this should be the case regardless of the type of filler (e.g., Pritchett, 1992). Unpublished work has demonstrated filled-gap effects for adjunct *wh*-phrases (Yoshida and Dickey, 2008), and recent work suggests an increase in processing difficulty associated for sentences with adjunct *wh*-phrases compared to sentences with no filler-gap dependency, implying active search processes are used even for adjunct filler-gap dependencies (Stepanov and Stateva, 2015).

Finally, another possible confound in our results is that Experiment 1 had rich contexts presented before the target item, but Experiments 2 and 3 did not. It is possible that this may have impacted the results in some way. However, we balanced the materials such that both interpretations of the ambiguous *wh*-question were pragmatically plausible. Additionally, findings from the Question After Story task in Omaki et al. (2013) English and Japanese converge with on-line findings in similarly context-less reading tasks (Aoshima et al., 2004). Thus, we tentatively take the results from the Question after Story task to reveal the same biases in filler-gap dependency processing as are observed in reading context-less sentences. However, it remains possible that context plays a greater role in Bangla than it does in prior Japanese studies.

## Conclusion

Much work in theoretical linguistics and psycholinguistics demonstrates that there are robust locality constraints on gaps. Both structural locality and linear locality play important roles in selecting gaps in real-time sentence processing. Structural locality is relevant for determining which gap sites are grammatically well-formed, and linear locality is relevant for determining which gaps are preferred when multiple potential gaps are available (Aoshima et al., 2004; Omaki et al., 2013). Locality biases on filler-gap dependencies can reveal themselves in different ways—as a general preference for certain gap sites, or as an early commitment. Typically, these are taken to reflect the same processes of active dependency formation, but different measures show that they dissociate. We investigated the dissociation of linear locality and structural locality by manipulating the flexible word order of Bangla, which allows testing the contribution of structural and linear locality.

In Experiment 1, we showed that Bangla speakers have a preference for linearly local gaps, regardless of structural position. This replicated findings from a previous English and Japanese comparison within the same language (Omaki et al., 2013), and thus supports the generalization that filler-gap dependency locality preferences are primarily sensitive to linear locality. However, in Experiments 2 and 3, we found evidence for filled-gap effects only when the disrupted position was both the linearly first position in the sentence and structurally closest. We highlighted a few reasons why this difference between word orders might hold. Specifically, we suggested that gaps in pre-verbal embedded clauses may be difficult to maintain, because of the status of pre-verbal embedded clauses in Bangla. Alternatively, we suggested that the informativity of the case system is such that comprehenders may not have a commitment to a gap position in place before the filled-gap region in the embedded verb first word orders. These facts contrast with Japanese, which exhibits a strong bias for gaps in pre-verbal embedded clauses (Aoshima et al., 2004; Yoshida, 2006; Omaki et al., 2013). If the results from Experiments 2 and 3 are amenable to these kinds of explanations, then it may be possible to retain the hypothesis that linear locality determines preferred gap sites in filler-gap dependency processing, whereas structural locality determines acceptable gap sites.

## Author contributions

DC: primary author, designed and conducted experiments; MI: primary author, designed and conducted experiments; SD: secondary author, designed and conducted experiments; SM: secondary author, designed experiments; MD: secondary author, designed experiments; CP: secondary author, designed experiments.

### Conflict of interest statement

The authors declare that the research was conducted in the absence of any commercial or financial relationships that could be construed as a potential conflict of interest.
