# Supplementary material for: Locality and Word Order in Active Dependency Formation in Bangla
Source: Front Psychol. 2016 Aug 25;7:1235. doi: 10.3389/fpsyg.2016.01235 (PMC4997089; doi:10.3389/fpsyg.2016.01235)
Supplement: Supplementary file 2 [file DataSheet1.pdf]

Supplementary Material

Locality and Word Order in Active Dependency Formation in Bangla

Dustin Alfonso Chacón*, Mashrur Imtiaz, Sikder Monoare Murshed, Sirsho Dasgupta, Mina Dan, Colin Phillips

*** Correspondence:** Corresponding Author: dustin.alfonso@nyu.edu

1 Materials for Experiment 1

Scripts for Experiment 1 videos:

Shumon:

সুমন একজন মহাকাশচারী। তিনি ভিনগ্রহেরপ্রাণী খুঁজে বেড়াচ্ছেন।সুমন যখন পৃথিবীতে ছিলেন তখন তিনি ভিনগ্রহেরপ্রাণীদের কোথায় পাওয়া যেতে পারে সে সম্বন্ধে আরও জানতে চেয়েছিলেন। কিন্তু তিনি তার অভিযানের প্রস্তুতি নিয়ে খুবই ব্যস্ত ছিলেন।যখন তিনি যাবার জন্য পুরোপুরি প্রস্তুত হলেন তখুনি তিনি রওনা দিলেন। ভিনগ্রহেরপ্রাণীদের ওপর গবেষণার জন্য তাঁর হাতে একটুও সময় রইলো না। নিজে কোনো গবেষণা করতে পারেন নি বলে তিনি ভাবলেন যে তাঁর বিজ্ঞানী বন্‌ধু যে মহাকাশে থাকে তাকে জিগ্যাসা করবেন। সুমন সেই বিজ্ঞানীর বন্‌ধুর সঙ্গে দেখা করতে কৃত্রিম উপগ্রহে গেলেন। সুমন তার সমস্ত পরামর্শ নিয়ে তার নিজের কোথায় যাওয়া উচিত সে সম্বন্ধে সিদ্ধান্ত নিলেন। তিনি বিজ্ঞানীকে বললেন, “ভিনগ্রহেরপ্রাণী খুঁজতে আমি মঙ্গলগ্রহে যাচ্ছি!” বিঞ্জানীও সায় দিয়ে বললেন যে এই পরিকল্পনাটা দারুণ, আর সুমনও তার দুঃসাহসিক অভিযানে বেরিয়ে পড়লেন। মঙ্গলে যাবার পথে তিনি চাঁদে থামলেন, ভাবলেন যদি সেখানেও তিনগ্রহেরপ্রাণী পাওয়া যায়। সর্বত্র খুঁজলেন। কিন্তু কোনো ভিনগ্রহেরপ্রাণী না পেয়ে আবার মঙ্গলের দিকে যাত্রা করলেন। মঙ্গলে পৌঁছে সুমন অবশেষে ভিনগ্রহেরপ্রাণীদের দেখতে পেলেন। তারা খুবই বন্‌ধুভাবাপন্ন আর এইরকম চমতকার ভিনগ্রহেরপ্রাণীদের সঙ্গে দেখা করতে পেরে, সুমন খুবই খুশি হলেন।

Polash:

পলাশ আজ স্কুলে থুবই ব্যস্ত সময় কাটিয়েছে এবং তার খুব ক্ষুধাও পেয়েছিলো। সে বাড়ির ফেরার পরে দেখলো যে তার ঘরে একটুকরো কেক রয়েছে। তার মনে পড়লো যে তার মা ও নানী মিলে সকালে একটি কেক বানিয়েছিলেন। ভালোই তো! কিন্তু কেকটা খেতে যাওয়ার সময় সে একটা খেলনার উপর হোঁচট খেলো এবং কেকটি মেঝেতে পড়ে গেলো। দুরছাই। পলাশ কেকটা খেতে পারলো না। কিন্তু সে তখনও ক্ষুধার্ত ছিলো। তাই সে আরও কেক আছে কিনা তা খুঁজতে গেলো। সে রান্না ঘরে দেখলো আরো কেক আছে এবং সে সেখান থেকে একটুকরো কেক খেয়ে ফেলো। যে কেকটা তার মা ও নানী বানিয়েছিলেন সেটা খুবই সুস্বাদু ছিলো। কেকটা শেষ করে সে বাথ্‌রুমে গেলো তার মাকে বলতে যে কেকটা খুব সুস্বাদু হয়েছে। কিন্তু সে কথা পলাশ তার মাকে বলতে পারলো না, কারণ তার মা ফোনে কথা বলছিলেন। সে কিছুক্ষণ অপেক্ষা করলো। কিন্তু মা ফোনে কথা বলেই যাচ্ছিলেন এবং তার দিকে কোনো মনোযোগই দিচ্ছিলেন না। পলাশের মন খারাপ হয়ে

গেলো, কারণ সে তার মাকে বলতে পারলো না যে কেকটা কত সুস্বাদু হয়েছিলো। কিন্তু তার মনে পড়লো যে তার নানী তার মাকে কেকটা বানাতে সাহায্য করেছিলেন। তাই সে ঠিক করলো পাশের বাড়িতে তার নানীর কাছে গিয়ে কেকটা কত মজার হয়েছে সেটা জানাবে। পলাশ নানীর বাড়িতে গিয়ে নানীকে বললো "আমার ঘরের কেকট পড়ে গিয়েছিলো, কিন্তু পরে আমি রান্না ঘরে গিয়ে কেকটার আরএকটুকরো নিয়ে খেলাম। কেকটা খুবই মজার হয়েছে।" পলাশ খুব খুশি যে সে তার নানীকে জানাতে পারলো যে কেকটা সুস্বাদু ছিলো। এটা শুনে পলাশের নানী খুবই খুশি হলেন।

Shumi:

সেটা ছিলো বসন্তের এক সুন্দর দিন। সেজন্যে সুমি ঠিক করলো যে সে পার্কে গিয়ে কয়েকটি প্রজাপতি ধরবে। তার মা-বাবা বাড়িতে ছিলেন না, সেজন্যে সুমি ভাবলো যে তার ভাই বা বোনকে পার্কে যাবার কথা জানান উচিত যাতে মা-বাবা ফিরে আসলে তাঁরা জানবেন সে কোথায়। প্রথমে সে তার ভাইয়ের ঘরে গেলো, কিন্তু সে দেখলো যে তার ভাই ঘুমচ্ছে। তাই সুমি তাকে প্রজাপতি ধরার কথা বলতে পারলো না। এর পর সে তার বোনকে খুঁজতে গেল। সে বাড়ির সবখানে খুঁজে দেখলো কিন্তু বোনকে কোথাও পেলো না। যখন সে হাল ছেড়ে দিচ্ছিল তখন নীচ তলা থেকে তার বোনের গলা শুনতে পেলো। সে নীচ তলায় গিয়ে বোনকে বললো "আমি পার্কে প্রজাপতি ধরতে যাচ্ছি।" সুমি পার্কে যাবার পথে গাড়ির পার্কিং স্থানে একটা প্রজাপতি দেখলো। সে আস্তে আস্তে প্রজাপতির দিকে হাঁটতে লাগলো। কিন্তু সুমি সেখানে পৌঁছানোর আগেই আরেকটা মেযে এসে প্রজাপতিটি ধরে ফেললো। সুমি সেখানে আর কোনো প্রজাপতি দেখলো না, তাই সে পার্কে ঢুকে গেলো। পার্কে অনেক প্রজাপতি দেখলো।সে একটি ধরে বয়ামে রাখলো এবং বাড়িতে ফিরে গেলো। সে য়ে প্রজাপতিটা ধরেছিলো সেটা তার খুবই পছন্দের। কিন্তু তার মনে হলো য়দি সে আরো প্রজাপতি ধরতে পারতো।

Sakib:

সাকিব ও তার মা গতকাল কেনাকাটা করতে গিয়ে একটা নতুন ফুটবল কিনেছিলো। তাই সাকিব আজ স্কুলের পরে ফুটবলটা নিযে খেলতে যাচ্ছিলো। স্কুলে সে তার বন্ধুদেরকে ফুটবল খেলবার কথা বলতে চাচ্ছিলো। সে জানতে চাচ্ছিলো য়ে তারা তার সাথে ফুটবল খেলতে চায় কিনা। কিন্তু সাকিব চায় নি য়ে তার শিক্ষক ক্লাসে কথা বলার জন্য তার উপর রাগ করেন। সে জন্য স্কুলে সে কিছুই বলেনি। সাকিব জানত যে স্কুলের পরে বাড়ি ফিরতে দেরী হলে মা চিন্তা করবেন। তাই সে মাকে তার পরিকল্পনার কথা বলতে গেলো। সে মাকে বললো, "আমি মাঠে ফুটবল খেলতে যাব।" তার মা খুশি হলেন যে সে ইতোমধ্যেই নতুন ফুটবলটা নিযে খেলা শুরু করেছে। ফুটবল খেলার মাঠে যাওয়ার পথে সে একটা পার্কের পাশ দিয়ে যাচ্ছিলো। সেখানে সে তার কয়েকজন বন্ধুর দেখা পেলো। সে চাইলো তারা যেন তার সাথে ফুটবল খেলে। কিন্তু তারা তখন ঘুড়ি উড়াচ্ছিলো, তাই তারা সাকিবের সাথে খেলার কোনো আগ্রহ দেখালো না। কিন্তু সাকিবের সবচেয়ে ঘণিষ্ট বন্ধু ঠিক করলো যে সে ঘুড়ি উড়ানো ছেড়ে সাকিবের সাথে ফুটবল খেলবে। তারা দুইজনে মিলে ফুটবলের মাঠে ফুটবল খেললো এবং সাকিব নতুন ফুটবল দিয়ে অনেক গোল করলো।

Protiti:

প্রতীতি ঘোড়ায় চড়া শিখছে। আজ সে একা একা ঘোড়ায় চড়ে অনেক দূর গেলো। প্রথমে সে পাহাড়ে উঠলো। কিন্তু পাহাড়ের পথটি ঘোড়ার জন্য খুব বেশি খাড়া ছিলো। তাই সে ঘোড়া নিয়ে সেখানে উঠতে পারলো না। বরং, প্রতীতি তার ঘোড়ায় চড়ে বনে গেলো। বনে ঘোড়ায় চড়ে বেরানো খুব

মজার ছিলো। প্রতীতি যেতে যেতে তার ঘোড়া দিয়ে পথের উপর থাকা একটি গুড়ি দিঙিয়ে গেলো। সে একা একা বনে ঘুরে বেড়াতে পেরেছে সে জন্যে খুবই আনন্দ বোধ করলো। সে তার ঘোড়ায় চড়ার কথা শিক্ষককে বলতে চাইলো। কিন্তু তার শিক্ষক অন্য আর একজন ছাত্রকে শেখাতে ব্যস্ত ছিলেন। সে জন্য প্রতীতি তার সাথে কথা বলতে পারলো না। সেই রাতে প্রতীতি ও তার এক বন্ধু ক্যাম্পিঙে গেলো। প্রতীতির বন্ধুও ঘোড়ায় চড়া শিখছিলো। তাই সে প্রতীতির কাছে তার ঘোড়ায় চড়া কেমন যাচ্ছে জানতে চাইলো। প্রতীতি তাকে বললো "আজকে আমি ঘোড়ায় চড়ে বনে বেড়িয়েছি। একটা বড়ো গুড়ির ওপর দিঙিয়েও গিয়েছি।" প্রিতীতির বন্ধু ঈর্ষান্বিত হলো যে প্রতীতি একা একা ঘোড়ায় করে বনে বেড়িয়েছে।

Sharmin:

শার্মীন ঘরের বাইরে খেলতে পছন্দ করে। এক দিন সে একটা দোলনায় দুলছিলো।সে দোলনার খুব উঁচু থেকে লাফ দিয়েছিলো। মাটিতে পড়ার আগে সে খুব ভালো করে ভারসাম্য বজা রাখলো। সে জন্যে পড়ে যায় নি। শার্মীন যখন দোলনায় নিয়ে খেলতে খেলতে বিরক্ত হয়ে গেলো, তখন সে ঠিক করলো যে একটা খুব উঁচু গাছে চড়বে। সে বেশ উঁচুতে উঠলো, কিন্তু হঠাত একটা ডাল ভেঙিয়ে গিয়ে গাছ থেকে পড়ে গেলো আর ব্যথাও পেলো। কিন্তু শারমীন বেশ সাহসী মেয়ে, সে জন্যে সে তখনই উঠে পড়লো আর একটু কাঁদলো না। শারমীন তার বন্ধুদের কাছে গর্ব করতে চাইলো যে সে পড়ে গিয়ে ব্যথা পাবার পরেও একটু কাঁদে নি। তাই সে লাইব্রেরিতে তার বন্ধুদেরকে খুঁজতে গেলো। সে তার এক বন্ধু খুঁজে পেলো, কিন্তু লাইব্রেরিয়ান এসে তাকে বললো যে লাইব্রেরিতে কথা বলা নিষেধ। সে জন্যে শারমীন তার বন্ধুর সাথে কথা বলতে পারলো না। তার মন খারাপ হয়ে গেলো। কিন্তু তার মাথায় এক বুদ্ধি এলো। সুইমিং পুলে তার আরো বন্ধুর আছে।সে তাদের সাথে দেখা করতে পারে কারণ সুইমিং পুলে যত ইচ্ছা তত কথা বলতে পারবে। সুইমিং পুলে পৌঁছিয়ে তার বন্ধুদেরকে খুঁজে পেয়ে বললো গাছ থেকে পড়ে আমি ব্যথা পেয়েছি, কিন্তু একটুও কাঁদিনি। শারমীন খুশি যে সে শেষ পর্যন্ত সে তার দিনের ঘটনাগুলোর কাউকে বলতে পারলো। এবং তার বন্ধুরা অবাক হলো তার সহাস দেখে।

Rupa:

এক দিন রূপা গুপ্তধন খুঁজে বের করার উপর একটি সিনেমা দেখে ভাবলো যে সে নিজে যদি গুপ্তধন খুঁজতে চায় তা হলে নাজানি কত মজা হবে। কিন্তু প্রথমে তার পরামর্শ দরকার যে সে কোথায় গুপ্তধন খুঁজে পাবে। সে বিশ্ববিদ্যালয়ের এক অধ্যাপকের কাছে পরামর্শ চাইতে গেলো যে কোথায় কোথায় গুপ্তধন খোঁজা যায়। কিন্তু অধ্যাপকের সাথে দেখা হলো না কেন না তিনি ছুটিতে গিয়েছেন। এর পর তার মনে হলো যে জাদুঘরের গাইড হিসাবে যিনি কাজ করেন তিনি জেনে থাকতে পারেন যে গুপ্তধন কোথায় পাওয়া যায়। তাই সে জাদুঘরে গেলো সেই গাইডের সাথে কথা বলতে। জাদুঘরের গাইড গুপ্তধন কোথায় পাওয়া যেতে পারে সে বিষয়ে যা যা জানতেন সেটা রূপাকে বললেন। এর পর রূপা তাকে বললো "ধন্যবাদ, আমি না হয় একটা ভাঙা জাহাজে গুপ্তধন খুঁজতে যাবো।" গাইড মত দিলেন যে সেটাই ভালো হবে। তিনি রূপাকে এক ভাঙা জাহাজের খোঁজ বের করে দিলেন। ভাঙা জাহাজে যাওয়ার সময় রূপা একটা গুহা দেখতে পেলো। সে ভাবলে যে গুহাতেও গুপ্তধন থাকতে পারে। তাই সে গুহার ভিতর যাওয়ার সিদ্ধান্ত নিলো। কিন্তু গুহাটিতে ঘুটঘুটে অন্ধকার আর সে জন্য সে কিছুই দেখতে পারছিলো না। রূপা খুবই ভয় পেয়ে গেলো। সে

তখনই গুহা থেকে বের হয়ে এলো এবং ভাঙা জাহাজের সন্ধানে গেলো। সে সাগরের জায়গা মতো পৌঁছালো। এর পর গগলস পরে ডুব দিয়ে ভাঙা জাহাজটির ভিতরে ঢুকলো। সে চারদিতে খুব ভালো করে খুঁজলো। অবশেষে সে একটি গুপ্তধন ভরা একটি সিন্ধুক খুঁজে পেলো। রূপা খুবই উত্তেজিত হয়ে গেলো এবং সে ঠিক করলো যে জাদুঘরের গাইডকে গুপ্তধনের ভাগ দেবে কারণ তার সাহায্য ছাড়া সে গুপ্তধন খুঁজে পেত না।

Oru:

অরু বিমান খুব পছন্দ করে। তার সবসময় বিমানের চালকের আসনে বসার খুব আগ্রহ। একবার যখন তার মা-বাবার সাথে বিমানবন্দরে এলো, তার ইচ্ছা হলো লুকিয়ে লুকিয়ে বিমানের ভিতর ঢুকে চালকের আসন দেখবে। কিন্তু বিমানের কাছাকাছে যাওয়ার আগেই একজন পুলিশ তাকে থামিয়ে দিলো। কারণ এই ভাবে যাওয়া খুব বিপজ্জনক। অরুর মন খারাপ হয়ে গেলো কারণ সে বিমানের ভিতরে যেতে পারলো না। তাই সে তার বাড়ির কাছের একটি বিমানের জাদুঘরে গেলো। বিমানের জাদুঘরে একটা পুরাণো বিমান ছিলো এবং তারা অরূকে চালকের আসনে বসতে দিলো। তার মনে হলো যে সে সত্যিকার একজন বিমান চালক। আর অরুর এত ভাল লাগলো যে সে ঠিক করলো বড়ো হয়ে বিমান চালক হবে। অরু খুব খুশি হয়ে বাড়ি গেলো এবং ভাবলো সে তার মা-বাবাকে বিমান চালক হবার নতুন স্বপ্নের কথা বলবে। কিন্তু তার মনে হলো যে তাঁরা এটাকে বিপজ্জনক পেশা মনে করে বাধা দেবেন। সে জন্য অরু তাদেরকে তার স্বপ্নের কথা না বলা সিদ্ধান্ত নিলো। পরের দিন স্কুলে গিয়ে সে বিঞ্জানের প্রিয় শিক্ষককে তার নতুন স্বপ্নের কথা বললো। সে তার শিক্ষককে বললো "আমি জাদুঘরে একটা বিমান চালকের আসনে বসে ছিলাম। আর এখন আমি বিমান চালক হতে চাই।" সব শুনে শিক্ষক খুবই খুশি হলেন. তিনি বিমান চালক হবার জন্য যা যা করতে হবে তা তাকে বলে দিলেন।

Stimuli for Experiment 1:
(main verb first; embedded verb first)

Shumon:

সুমন কোথায় একজনকে বলেছেন যে তিনি ভিনগ্রহেরপ্রাণী দেখবেন?
সুমন কোথায় তিনি ভিনগ্রহেরপ্রাণী দেখবেন বলে একজনকে বলেছেন?

Polash:

পলাশ কোথায় একজনকে বলেছে যে সে একটুকরো কেক খেয়েছে?
পলাশ কোথায় সে একটুকরো কেক খেয়েছে বলে একজনকে বলেছে?

Shumi:

সুমি কোথায় একজনকে বলেছে যে সে প্রজাপতি ধরতে চায়?
সুমি কোথায় সে প্রজাপতি ধরতে চায় বলে একজনকে বলেছে?

Sakib:

সাকিব কোথায় একজনকে বলেছে যে সে ফুটবল খেলবে?
সাকিব কোথায় সে ফুটবল খেলবে বলে একজনকে বলেছে?

Protiti:

প্রতীতি কোথায় একজনকে বলেছে যে সে ঘোড়ায় চড়বে?
প্রতীতি কোথায় সে ঘোড়ায় চড়বে বলে একজনকে বলেছে?

Sharmin:

শারমিন কাথোয় একজনকে বলেছে যে সে ব্যথা পেয়েছে?
শারমিন কোথায় সে ব্যথা পেয়েছে বলে একজনকে বলেছে?

Rupa:
রূপা কোথায় একজনকে বলেছে যে সে গুপ্তধন খুঁজে পাবে?
রূপা কোথায় গুপ্তধন খুঁজে পাবে বলে একজনকে বলেছে?
Oru:
অরু কোথায় একজনকে বলেছে যে সে বিমান চালকের আসনে বসে ছিলো?
অরু কোথায় সে বিমান চালকের আসনে বসে ছিলো বলে একজনকে বলেছে?

2 Materials for Experiment 2

a, 1 রাশাদ জিজ্ঞাসা করেছে কাকে ডাক্তারটা খুবই অবাকভাবে রুগীকে বলেছেন যে তিনি পুরানো হাসপাতালে চিকিৎসা করেছেন

b, 1 রাশাদ জিজ্ঞাসা করেছে কখন ডাক্তারটা খুবই অবাকভাবে রুগীকে বলেছেন যে তিনি পুরানো হাসপাতালে তাকে চিকিৎসা করেছেন

c, 1 রাশাদ জিজ্ঞাসা করেছে কাকে ডাক্তারটা তিনি পুরানো হাসপাতালে রুগীকে চিকিৎসা করেছেন বলে খুবই অবাকভাবে বলেছেন

d, 1 রাশাদ জিজ্ঞাসা করেছে কখন ডাক্তারটা তিনি পুরানো হাসপাতালে রুগীকে চিকিৎসা করেছেন বলে খুবই অবাকভাবে তাকে বলেছেন

a, 2 জাহিদ পত্রিকায় পড়েছে কাকে রাজনীতবিদটা বেশ জোরে সাংবাদিকটাকে বলেছেন যে তিনি জাতিসংঘের ভবনে অভিবাদন জানিয়েছেন

b, 2 জাহিদ পত্রিকায় পড়েছে কখন রাজনীতবিদটা বেশ জোরে সাংবাদিকটাকে বলেছেন যে তিনি জাতিসংঘের ভবনে তাকে অভিবাদন জানিয়েছেন

c, 2 জাহিদ পত্রিকায় পড়েছে কাকে রাজনীতবিদটা তিনি জাতিসংঘের ভবনে সাংবাদিকটাকে অভিবাদন জানিয়েছেন বলে বেশ জোরে বলেছেন

d, 2 জাহিদ পত্রিকায় পড়েছে কখন রাজনীতবিদটা তিনি জাতিসংঘের ভবনে সাংবাদিকটাকে অভিবাদন জানিয়েছেন বলে বেশ জোরে তাকে বলেছেন

a, 3 সীতা শুনেছে কাকে বিজ্ঞানীটা কিছুটা বিস্মিতভাবে গবেষককে বলেছেন যে তিনি বার্ষিক অনুষ্ঠানটাতে পুরষ্কার দেবেন

b, 3 সীতা শুনেছে কখন বিজ্ঞানীটা কিছুটা বিস্মিতভাবে গবেষককে বলেছেন যে তিনি বার্ষিক অনুষ্ঠানটাতে তাকে পুরষ্কার দেবেন

c, 3 সীতা শুনেছে কাকে বিজ্ঞানীটা তিনি বার্ষিক অনুষ্ঠানটাতে গবেষককে পুরষ্কার দেবেন বলে কিছুটা বিস্মিতভাবে বলেছেন

d, 3 সীতা শুনেছে কখন বিজ্ঞানীটা তিনি বার্ষিক অনুষ্ঠানটাতে গবেষককে পুরষ্কার দেবেন বলে কিছুটা বিস্মিতভাবে তাকে বলেছেন

a, 4 জোনাকী ঘোষণা করবে কাকে ব্যবসায়ীটা খানিকটা সাবধানে বিনিয়োগকারীকে বলেছেন যে তিনি নতুন কোম্পানীটাতে চাকরি দেবেন

b, 4 জোনাকী ঘোষণা করবে কখন ব্যবসায়ীটা খানিকটা সাবধানে বিনিয়োগকারীকে বলেছেন যে তিনি নতুন কোম্পানীটাতে তাকে চাকরি দেবেন

c, 4 জোনাকী ঘোষণা করবে কাকে ব্যবসায়ীটা তিনি নতুন কোম্পানীটাতে বিনিয়োগকারীকে চাকরি দেবেন বলে খানিকটা সাবধানে বলেছেন

d, 4 জোনাকী ঘোষণা করবে কখন ব্যবসায়ীটা তিনি নতুন কোম্পানীটাতে বিনিয়োগকারীকে চাকরি দেবেন বলে খানিকটা সাবধানে তাকে বলেছেন

a, 5 সাবরিনা এখনও জানে কাকে শিক্ষকটা অত্যন্ত সহজভাবে ছাত্রটাকে বলেছেন যে তিনি গ্রামের স্কুলটাতে শেখাতে চান

b, 5 সাবরিনা এখনও জানে কখন শিক্ষকটা অত্যন্ত সহজভাবে ছাত্রটাকে বলেছেন যে তিনি গ্রামের স্কুলটাতে তাকে শেখাতে চান

c, 5 সাবরিনা এখনও জানে কাকে শিক্ষকটা তিনি গ্রামের স্কুলটাতে ছাত্রটাকে শেখাতে চান বলে অত্যন্ত সহজভাবে বলেছেন

d, 5 সাবরিনা এখনও জানে কখন শিক্ষকটা তিনি গ্রামের স্কুলটাতে ছাত্রটাকে শেখাতে চান বলে অত্যন্ত সহজভাবে তাকে বলেছেন

a, 6 এনাম সিদ্ধান্ত নিয়েছে কাকে উকিলটা অনেকটা সোজাসুজিভাবে বন্দীকে বলেছেন যে তিনি আঞ্চলিক আদালতটাতে রক্ষা করবেন

b, 6 এনাম সিদ্ধান্ত নিয়েছে কখন উকিলটা অনেকটা সোজাসুজিভাবে বন্দীকে বলেছেন যে তিনি আঞ্চলিক আদালতটাতে তাকে রক্ষা করবেন

c, 6 এনাম সিদ্ধান্ত নিয়েছে কাকে উকিলটা তিনি আঞ্চলিক আদালতটাতে বন্দীকে রক্ষা করবেন বলে অনেকটা সোজাসুজি বলেছেন

d, 6 এনাম সিদ্ধান্ত নিয়েছে কখন উকিলটা তিনি আঞ্চলিক আদালতটাতে বন্দীকে রক্ষা করবেন বলে অনেকটা সোজাসুজি তাকে বলেছেন

a, 7 নিপা জানতে চাচ্ছিলো কাকে পুলিসটি কিছুটা বিষণ্ণভাবে অপরাধের_শিকারটাকে বলেছেন যে তিনি আপরাধের জন্য শাস্তি দেবেন

b, 7 নিপা জানতে চাচ্ছিলো কখন পুলিসটি কিছুটা বিষণ্ণভাবে অপরাধের_শিকারটাকে বলেছেন যে তিনি আপরাধের জন্য তাকে শাস্তি দেবেন

c, 7 নিপা জানতে চাচ্ছিলো কাকে পুলিসটি তিনি আপরাধের জন্য অপরাধের_শিকারটাকে শাস্তি

দেবেন বলে কিছুটা বিষণ্ণভাবে বলেছেন

d, 7 নিপা জানতে চাচ্ছিলো কখন পুলিসটি তিনি আপরাধের জন্য অপরাধের_শিকারটাকে শাস্তি দেবেন বলে কিছুটা বিষণ্ণভাবে তাকে বলেছেন

a, 8 সেনিয়া আবিষ্কার করেছে কাকে দোকানিটা খুব অপ্রত্যাশিতভাবে খরিদ্দারটাকে বলেছেন যে তিনি ফলের দোকানটাতে কাঠাল কিনে_দিয়েছেন

b , 8 সেনিয়া আবিষ্কার করেছে কখন দোকানিটা খুব অপ্রত্যাশিতভাবে খরিদ্দারটাকে বলেছেন যে তিনি ফলের দোকানটাতে তাকে কাঠাল কিনে_দিয়েছেন

c, 8 সেনিয়া আবিষ্কার করেছে কাকে দোকানিটা তিনি ফলের দোকানটাতে খরিদ্দারটাকে কাঠাল কিনে_দিয়েছেন বলে খুব অপ্রত্যাশিতভাবে বলেছেন

d, 8 সেনিয়া আবিষ্কার করেছে কখন দোকানিটা তিনি ফলের দোকানটাতে খরিদ্দারটাকে কাঠাল কিনে_দিয়েছেন বলে খুব অপ্রত্যাশিতভাবে তাকে বলেছেন

a, 9 সুমী শিখেছে কাকে অনুবাদকটা কিছুটা বিষণ্ণভাবে কবিকে বলেছেন যে তিনি শহরের লাইব্রেরীটাতে কবিতা দেবেন_না

b, 9 সুমী শিখেছে কখন অনুবাদকটা কিছুটা বিষণ্ণভাবে কবিকে বলেছেন যে তিনি শহরের লাইব্রেরীটাতে তাকে কবিতা দেবেন_না

c, 9 সুমী শিখেছে কাকে অনুবাদকটাতিনি শহরের লাইব্রেরীটাতে কবিকে কবিতা দেবেন_না বলে কিছুটা বিষণ্ণভাবে বলেছেন

d, 9 সুমী শিখেছে কখন অনুবাদকটাতিনি শহরের লাইব্রেরীটাতে কবিকে কবিতা দেবেন_না বলে কিছুটা বিষণ্ণভাবে তাকে বলেছেন

a, 10 সালমা জানতো না কাকে লেখকটা বেশ তাড়াতাড়ি প্রকাশকটাকে বলেছেন যে তিনি আন্তর্জাতিক সভাটাতে সাহায্য করবেন

b, 10 সালমা জানতো না কখন লেখকটা বেশ তাড়াতাড়ি প্রকাশকটাকে বলেছেন যে তিনি আন্তর্জাতিক সভাটাতে তাকে সাহায্য করবেন

c, 10 সালমা জানতো না কাকে লেখকটাতিনি আন্তর্জাতিক সভাটাতে প্রকাশকটাকে সাহায্য করবেন বলে বেশ তাড়াতাড়ি বলেছেন

d, 10 সালমা জানতো না কখন লেখকটাতিনি আন্তর্জাতিক সভাটাতে প্রকাশকটাকে সাহায্য করবেন বলে বেশ তাড়াতাড়ি তাকে বলেছেন

a, 11 তানভীর জিজ্ঞাসা করেছে কাকে প্রকৌশলীটা খুব ভদ্রভাবে স্থপতিটাকে বলেছেন যে তিনি বাড়িটার জন্য অনুরোধ করবেন

b, 11 তানভীর জিজ্ঞাসা করেছে কখন প্রকৌশলীটা খুব ভদ্রভাবে স্থপতিটাকে বলেছেন যে তিনি বাড়িটার জন্য তাকে অনুরোধ করবেন

c, 11 তানভীর জিজ্ঞাসা করেছে কাকে প্রকৌশলীটা তিনি বাড়িটার জন্য স্থপতিটাকে অনুরোধ করবেন বলে খুব ভদ্রভাবে বলেছেন

d,11 তানভীর জিজ্ঞাসা করেছে কখন প্রকৌশলীটা তিনি বাড়িটার জন্য স্থপতিটাকে অনুরোধ করবেন বলে খুব ভদ্রভাবে তাকে বলেছেন

a, 12 আকাশ খবরের_কাগজে পড়েছে কাকে প্রধানমন্ত্রী খানিকটা সাবধানে গুপ্তচরটাকে বলেছেন যে তিনি একটা বিমানটাতে হত্যা করে_দেবেন

b, 12 আকাশ খবরের_কাগজে পড়েছে কখন প্রধানমন্ত্রী খানিকটা সাবধানে গুপ্তচরটাকে বলেছেন যে তিনি একটা বিমানটাতে তাকে হত্যা করে_দেবেন

c, 12 আকাশ খবরের_কাগজে পড়েছে কাকে প্রধানমন্ত্রী তিনি একটা বিমানটাতে গুপ্তচরটাকে হত্যা করে_দেবেন বলে খানিকটা সাবধানে বলেছেন

d, 12 আকাশ খবরের_কাগজে পড়েছে কখন প্রধানমন্ত্রী তিনি একটা বিমানটাতে গুপ্তচরটাকে হত্যা করে_দেবেন বলে খানিকটা সাবধানে তাকে বলেছেন

a, 13 সোমা শুনেছে কাকে পাচকটা অনেকটা গোপনে খাদকটাকে বলেছেন যে তিনি বড় রেস্টুরেন্টটাতে নিমন্ত্রণ করবেন

b, 13 সোমা শুনেছে কখন পাচকটা অনেকটা গোপনে খাদকটাকে বলেছেন যে তিনি বড় রেস্টুরেন্টটাতে তাকে নিমন্ত্রণ করবেন

c, 13 সোমা শুনেছে কাকে পাচকটা কাকে তিনি বড় রেস্টুরেন্টটাতে খাদকটাকে নিমন্ত্রণ করবেন বলে অনেকটা গোপনে বলেছেন

d, 13 সোমা শুনেছে কখন পাচকটা তিনি বড় রেস্টুরেন্টটাতে খাদকটাকে নিমন্ত্রণ করবেন বলে অনেকটা গোপনে তাকে বলেছেন

a, 14 জামাল ঘোষণা করেছে কাকে গায়কটা বেশ শান্তভাবে অভিনেত্রীটাকে বলেছেন যে তিনি সুন্দর থিয়েটারটাতে বিয়ে করবে

b, 14 জামাল ঘোষণা করেছে কখন গায়কটা বেশ শান্তভাবে অভিনেত্রীটাকে বলেছেন যে তিনি সুন্দর থিয়েটারটাতে তাকে বিয়ে করবে

c, 14 জামাল ঘোষণা করেছে কাকে গায়কটা বলে তিনি সুন্দর থিয়েটারটাতে অভিনেত্রীটাকে বিয়ে করবে বেশ শান্তভাবে বলেছেন
d, 14 জামাল ঘোষণা করেছে কখন গায়কটা বলে তিনি সুন্দর থিয়েটারটাতে অভিনেত্রীটাকে বিয়ে করবে বেশ শান্তভাবে তাকে বলেছেন

a, 15 জয় অনুমান করেছে কাকে ধোপাটা কিছুটা রাগতভাবে দাসীটাকে বলেছেন যে তিনি কাজের জন্য টাকা দেবেন_না

b, 15 জয় অনুমান করেছে কখন ধোপাটা কিছুটা রাগতভাবে দাসীটাকে বলেছেন যে তিনি কাজের জন্য তাকে টাকা দেবেন_না

c, 15 জয় অনুমান করেছে কাকে ধোপাটা তিনি কাজের জন্য দাসীটাকে টাকা দেবেন_না বলে কিছুটা রাগতভাবে বলেছেন

d, 15 জয় অনুমান করেছে কখন ধোপাটা তিনি কাজের জন্য দাসীটাকে টাকা দেবেন_না বলে কিছুটা রাগতভাবে তাকে বলেছেন

a,16 সজল সিদ্ধান্ত নিয়েছে কখন জেনারেল খুব ভোরে সৈনিকটাকে বলেছেন যে তিনি বিশাল যুদ্ধক্ষেত্রটাতে আক্রমণ করবেন

b, 16 সজল সিদ্ধান্ত নিয়েছে কখন জেনারেল খুব ভোরে সৈনিকটাকে বলেছেন যে তিনি বিশাল যুদ্ধক্ষেত্রটাতে তাকে আক্রমণ করবেন

c, 16 সজল সিদ্ধান্ত নিয়েছে কাকে জেনারেল তিনি বিশাল যুদ্ধক্ষেত্রটাতে সৈনিকটাকে আক্রমণ করবেন বলে খুব ভোরে বলেছেন

d, 16 সজল সিদ্ধান্ত নিয়েছে কখন জেনারেল তিনি বিশাল যুদ্ধক্ষেত্রটাতে সৈনিকটাকে আক্রমণ করবেন বলে খুব ভোরে তাকে বলেছেন

a,17 মিতা জানতে চাচ্ছিলো কাকে দর্জিটা প্রায় উত্তেজিতভাবে শিক্ষানবিশটাকে বলেছেন যে তিনি গ্রামের বাজারে দেখতে পারবেন

b, 17 মিতা জানতে চাচ্ছিলো কোথায় দর্জিটা প্রায় উত্তেজিতভাবে শিক্ষানবিশটাকে বলেছেন যে তিনি গ্রামের বাজারে তাকে দেখতে পারবেন

c, 17 মিতা জানতে চাচ্ছিলো কাকে দর্জিটা তিনি গ্রামের বাজারে শিক্ষানবিশটাকে দেখতে পারবেন বলে প্রায় উত্তেজিতভাবে বলেছেন

d, 17 মিতা জানতে চাচ্ছিলো কোথায় দর্জিটা তিনি গ্রামের বাজারে শিক্ষানবিশটাকে দেখতে পারবেন বলে প্রায় উত্তেজিতভাবে তাকে বলেছেন

a,18 নয়ন আবিষ্কার করেছে কাকে পাণ্ডিতটা খুব সহজভাবে ঋষিটাকে বলেছেন যে তিনি প্রাচীন মন্দিরটাতে নমস্কার করেছেন

b, 18 নয়ন আবিষ্কার করেছে কোথায় পাণ্ডিতটা খুব সহজভাবে ঋষিটাকে বলেছেন যে তিনি প্রাচীন মন্দিরটাতে তাকে নমস্কার করেছেন

c,18 নয়ন আবিষ্কার করেছে কাকে পাণ্ডিতটা তিনি প্রাচীন মন্দিরটাতে ঋষিটাকে নমস্কার করেছেন বলে খুব সহজভাবে বলেছেন

d,18 নয়ন আবিষ্কার করেছে কোথায় পাণ্ডিতটা তিনি প্রাচীন মন্দিরটাতে ঋষিটাকে নমস্কার করেছেন বলে খুব সহজভাবে তাকে বলেছেন

a, 19 আলম শিখেছে কাকে অধ্যাপকটা খুব কৌতূহলীভাবে দার্শনিকটাকে বলেছেন যে তিনি বই_মেলাটা থেকে বই কিনে_দেবে

b, 19 আলম শিখেছে কোথায় অধ্যাপকটা খুব কৌতূহলীভাবে দার্শনিকটাকে বলেছেন যে তিনি বই_মেলাটা থেকে তাকে বই কিনে_দেবে

c, 19 আলম শিখেছে কাকে অধ্যাপকটা তিনি বই_মেলাটা থেকে দার্শনিকটাকে বই কিনে_দেবে বলে খুব কৌতূহলীভাবে বলেছেন

d, 19 আলম শিখেছে কোথায় অধ্যাপকটা তিনি বই_মেলাটা থেকে দার্শনিকটাকে বই কিনে_দেবে বলে খুব কৌতূহলীভাবে তাকে বলেছেন

a, 20 রোকন জানতো না কাকে গোয়েন্দাটা খুব রহস্যজনকভাবে স্বেচ্ছাসেবকটাকে বলেছেন যে তিনি খুব রাতে অনুসন্ধান করবেন

b, 20 রোকন জানতো না কোথায় গোয়েন্দাটা খুব রহস্যজনকভাবে স্বেচ্ছাসেবকটাকে বলেছেন যে তিনি খুব রাতে তাকে অনুসন্ধান করবেন

c, 20 রোকন জানতো না কাকে গোয়েন্দাটা তিনি খুব রাতে স্বেচ্ছাসেবকটাকে অনুসন্ধান করবেন বলে খুব রহস্যজনকভাবে বলেছেন

d, 20 রোকন জানতো না কোথায় গোয়েন্দাটা তিনি খুব রাতে স্বেচ্ছাসেবকটাকে অনুসন্ধান করবেন বলে খুব রহস্যজনকভাবে তাকে বলেছেন

a, 21 শীলা জিজ্ঞাসা করেছে কাকে বিমানচালকটা বেশ হঠাৎ যাত্রীসেবিকাটাকে বলেছেন যে তিনি একদম আন্তরিকভাবে আদর করেন

b, 21 শীলা জিজ্ঞাসা করেছে কোথায় বিমানচালকটা বেশ হঠাৎ যাত্রীসেবিকাটাকে বলেছেন যে তিনি একদম আন্তরিকভাবে তাকে আদর করেন

c, 21 শীলা জিজ্ঞাসা করেছে কাকে বিমানচালকটা তিনি একদম আন্তরিকভাবে যাত্রীসেবিকাটাকে আদর করেন বলে বেশ হঠাৎ বলেছেন

d, 21 শীলা জিজ্ঞাসা করেছে কোথায় বিমানচালকটা তিনি একদম আন্তরিকভাবে যাত্রীসেবিকাটাকে আদর করেন বলে বেশ হঠাৎ তাকে বলেছেন

a, 22 মণীষা ইনটারনেটে পড়েছে কাকে ভ্রমণকারীটা খুবই অপ্রত্যাশিতভাবে পাহারাদারটাকে বলেছেন যে তিনি কলকাতার বিমানবন্দরটাতে ডাকাতি করেছেন

b, 22 মণীষা ইনটারনেটে পড়েছে কোথায় ভ্রমণকারীটা খুবই অপ্রত্যাশিতভাবে পাহারাদারটাকে

বলেছেন যে তিনি কলকাতার বিমানবন্দরটাতে তাকে ডাকাতি করেছেন

c, 22 মণীষা ইনটারনেটে পড়েছে কাকে ভ্রমণকারীটা তিনি কলকাতার বিমানবন্দরটাতে পাহারাদারটাকে ডাকাতি করেছেন বলে খুবই অপ্রত্যাশিতভাবে বলেছেন

d, 22 মণীষা ইনটারনেটে পড়েছে কোথায় ভ্রমণকারীটা তিনি কলকাতার বিমানবন্দরটাতে পাহারাদারটাকে ডাকাতি করেছেন বলে খুবই অপ্রত্যাশিতভাবে তাকে বলেছেন

a, 23 সায়েম রেডিওতে শুনেছে কাকে উকিলটা অনেক জোরালোভাবে ভোটতাদাটাকে বলেছেন যে তিনি গুরুত্বপুর্ণ নির্বাচনে সমর্থন করবেন

b, 23 সায়েম রেডিওতে শুনেছে কোথায় উকিলটা অনেক জোরালোভাবে ভোটতাদাটাকে বলেছেন যে তিনি গুরুত্বপুর্ণ নির্বাচনে তাকে সমর্থন করবেন

c, 23 সায়েম রেডিওতে শুনেছে কাকে উকিলটাতিনি গুরুত্বপুর্ণ নির্বাচনে ভোটতাদাটাকে সমর্থন করবেন বলে অনেক জোরালোভাবে বলেছেন

d, 23 সায়েম রেডিওতে শুনেছে কোথায় উকিলটাতিনি গুরুত্বপুর্ণ নির্বাচনে ভোটতাদাটাকে সমর্থন করবেন বলে অনেক জোরালোভাবে তাকে বলেছেন

a, 24 নাজিয়া ঘোষণা করেছে কাকে চাষিটা প্রায় নিশ্চিতভাবে তাঁতিটাকে বলেছেন যে তিনি আগামী সপ্তাহে হাতিয়ার দেবেন

b, 24 নাজিয়া ঘোষণা করেছে কোথায় চাষিটা প্রায় নিশ্চিতভাবে তাঁতিটাকে বলেছেন যে তিনি আগামী সপ্তাহে তাকে হাতিয়ার দেবেন

a, 24 নাজিয়া ঘোষণা করেছে কাকে চাষিটা তিনি আগামী সপ্তাহে তাঁতিটাকে হাতিয়ার দেবেন বলে প্রায় নিশ্চিতভাবে বলেছেন

d, 24 নাজিয়া ঘোষণা করেছে কোথায় চাষিটা তিনি আগামী সপ্তাহে তাঁতিটাকে হাতিয়ার দেবেন বলে প্রায় নিশ্চিতভাবে তাকে বলেছেন

a, 25 রূপা এখনও জানে কাকে ভাস্করটা বেশ উত্তেজিতভাবে চিত্রকরটাকে বলেছেন যে তিনি শিল্প যাদুঘরটাতে প্রসংসা করেছেন

b, 25 রূপা এখনও জানে কোথায় ভাস্করটা বেশ উত্তেজিতভাবে চিত্রকরটাকে বলেছেন যে তিনি শিল্প যাদুঘরটাতে তাকে প্রসংসা করেছেন

c, 25 রূপা এখনও জানে কাকে ভাস্করটাতিনি শিল্প যাদুঘরটাতেচিত্রকরটাকে প্রসংসা করেছেন বলে বেশ উত্তেজিতভাবে বলেছেন

d, 25 রূপা এখনও জানে কোথায় ভাস্করটাতিনি শিল্প যাদুঘরটাতেচিত্রকরটাকে প্রসংসা করেছেন বলে বেশ উত্তেজিতভাবে তাকে বলেছেন

a, 26 ললিতা অনুমান করেছে কাকে ভাড়াটেটা বেশ দুঃখিতভাবে জমিদারটাকে বলেছেন যে তিনি এই বছরে ভাড়া দেবেন_না

b, 26 ললিতা অনুমান করেছে কোথায় ভাড়াটেটা বেশ দুঃখিতভাবে জমিদারটাকে বলেছেন যে তিনি এই বছরে তাঁকে ভাড়া দেবেন_না

c, 26 ললিতা অনুমান করেছে কাকে ভাড়াটেটাতিনি এই বছরে জমিদারটাকে ভাড়া দেবেন_না বলে বেশ দুঃখিতভাবে বলেছেন

d, 26 ললিতা অনুমান করেছে কোথায় ভাড়াটেটাতিনি এই বছরে জমিদারটাকে ভাড়া দেবেন_না বলে বেশ দুঃখিতভাবে তাঁকে বলেছেন

a, 27 ইভা আবিষ্কার করেছে কাকে চিত্রনির্মাতাটি বেশ গোপনভাবে অভিনেতাটাকে বলছেন যে তিনি তাঁর স্টুডিওটায় ফিল্ম তুলবেন

b, 27 ইভা আবিষ্কার করেছে কোথায় চিত্রনির্মাতাটি বেশ গোপনভাবে অভিনেতাটাকে বলছেন যে তিনি তাঁর স্টুডিওটায় ফিল্ম তুলবেন

c, 27 ইভা আবিষ্কার করেছে কাকে চিত্রনির্মাতাটি তিনি তাঁর স্টুডিওটায় অভিনেতাটাকে ফিল্ম তুলবেন বলে বেশ গোপনভাবে বলছেন

d, 27 ইভা আবিষ্কার করেছে কোথায় চিত্রনির্মাতাটি তিনি তাঁর স্টুডিওটায় অভিনেতাটাকে ফিল্ম তুলবেন বলে বেশ গোপনভাবে তাকে বলছেন

a, 28 করিম কালকে শিখেছে কাকে হিসাবরক্ষকটা খুবই গম্ভীরভাবে প্রশাসকটাকে বলেছেন যে তিনি চাকরিটার জন্য সুপারিশ করবেন

b, 28 করিম কালকে শিখেছে কোথায় হিসাবরক্ষকটা খুবই গম্ভীরভাবে প্রশাসকটাকে বলেছেন যে তিনি চাকরিটার জন্য তাকে সুপারিশ করবেন

c, 28 করিম কালকে শিখেছে কাকে হিসাবরক্ষকটা তিনি চাকরিটার জন্য প্রশাসকটাকে সুপারিশ করবেন বলে খুবই গম্ভীরভাবে বলেছেন

d, 28 করিম কালকে শিখেছে কোথায় হিসাবরক্ষকটা তিনি চাকরিটার জন্য প্রশাসকটাকে সুপারিশ করবেন বলে খুবই গম্ভীরভাবে তাকে বলেছেন

a, 29 সামীরা জিজ্ঞাসা করেছে কাকে গায়কটা কিছুটা লাজুকভাবে বাউলটাকে বলেছেন যে তিনি অনেকদিন আগে রেকোর্ড করেছেন

b, 29 সামীরা জিজ্ঞাসা করেছে কোথায় গায়কটা কিছুটা লাজুকভাবে বাউলটাকে বলেছেন যে তিনি অনেকদিন আগে তাকে রেকোর্ড করেছেন

c, 29 সামীরা জিজ্ঞাসা করেছে কাকে গায়কটা তিনি অনেকদিন আগে বাউলটাকে রেকোর্ড করেছেন বলে কিছুটা লাজুকভাবে বলেছেন

d, 29 সামীরা জিজ্ঞাসা করেছে কোথায় গায়কটা তিনি অনেকদিন আগে বাউলটাকে রেকোর্ড করেছেন বলে কিছুটা লাজুকভাবে তাকে বলেছেন

a, 30 মনির বিবেচনা করেছে কাকে নৃতত্ত্ববিদটা অনেক গর্বিতভাবে গ্রামবাসীটাকে বলেছেন যে তিনি আর একবার পরামর্শ করবেন

b, 30 মনির বিবেচনা করেছে কোথায় নৃতত্ত্ববিদটা অনেক গর্বিতভাবে গ্রামবাসীটাকে বলেছেন যে তিনি আর একবার তাকে পরামর্শ করবেন

c, 30 মনির বিবেচনা করেছে কাকে নৃতত্ত্ববিদটা তিনি আর একবার গ্রামবাসীটাকে পরামর্শ করবেন বলে অনেক গর্বিতভাবে বলেছেন

d, 30 মনির বিবেচনা করেছে কোথায় নৃতত্ত্ববিদটা তিনি আর একবার গ্রামবাসীটাকে পরামর্শ করবেন বলে অনেক গর্বিতভাবে তাকে বলেছেন

a, 31 অজয় টি.ভি.তে দেখেছে কাকে বিচারপতিটা কয়েক বার অপরাধীটাকে বলেছেন যে তিনি বিচারের পরে নিন্দা করবেন

b, 31 অজয় টি.ভি.তে দেখেছে কোথায় বিচারপতিটা কয়েক বার অপরাধীটাকে বলেছেন যে তিনি বিচারের পরে তাকে নিন্দা করবেন

c, 31 অজয় টি.ভি.তে দেখেছে কাকে বিচারপতিটা তিনি বিচারের পরে অপরাধীটাকে নিন্দা করবেন বলে কয়েক বার বলেছেন

d, 31 অজয় টি.ভি.তে দেখেছে কোথায় বিচারপতিটা তিনি বিচারের পরে অপরাধীটাকে নিন্দা করবেন বলে কয়েক বার তাকে বলেছেন

a, 32 নায়রা বিবেচনা করেছে কাকে মডেলটা হাসি নিয়ে ফোটোগ্রাফারটাকে বলেছেন যে তিনি তাদের মহলায় জড়িয়ে ধরবেন

b, 32 নায়রা বিবেচনা করেছে কোথায় মডেলটা হাসি নিয়ে ফোটোগ্রাফারটাকে বলেছেন যে তিনি তাদের মহলায় তাকে জড়িয়ে ধরবেন

c, 32 নায়রা বিবেচনা করেছে কাকে মডেলটা তিনি তাদের মহলায় ফোটোগ্রাফারটাকে জড়িয়ে ধরবেন বলে হাসি নিয়ে বলেছেন

d, 32 নায়রা বিবেচনা করেছে কোথায় মডেলটা তিনি তাদের মহলায় ফোটোগ্রাফারটাকে জড়িয়ে ধরবেন বলে হাসি নিয়ে তাকে বলেছেন

3 Materials for Experiment 2

1 a জাহিদ কাকে খুবই অবাকভাবে তার বন্ধুকে বলেছে যে নিপা পার্টিতে দেখেছে?

1 b জাহিদ কখন খুবই অবাকভাবে তার বন্ধুকে বলেছে যে নিপা পার্টিতে দেখেছে?

1 c জাহিদ কাকে নিপা পার্টিতে তার বন্ধুকে দেখেছে বলে খুবই অবাকভাবে বলেছে?

1 d জাহিদ কখন নিপা পার্টিতে তার বন্ধুকে দেখেছে বলে খুবই অবাকভাবে বলেছে?

1 e জাহিদ কাকে খুবই অবাকভাবে বলেছে যে নিপা পার্টিতে তার বন্ধুকে দেখেছে?

1 f জাহিদ কখন খুবই অবাকভাবে বলেছে যে নিপা পার্টিতে তার বন্ধুকে দেখেছে?

1 g জাহিদ কাকে নিপা পার্টিতে দেখেছে বলে খুবই অবাকভাবে তার বন্ধুকে বলেছে?

1 h জাহিদ কখন নিপা পার্টিতে দেখেছে বলে খুবই অবাকভাবে তার বন্ধুকে বলেছে?

2 a সাবরিনা কাকে বেশ জোরে তার বাবাকে বলেছে যে সেনিয়া তার অফিসে ছবিটা দেখিয়েছে?

2 b সাবরিনা কখন বেশ জোরে তার বাবাকে বলেছে যে সেনিয়া তার অফিসে ছবিটা দেখিয়েছে?

2 c সাবরিনা কাকে সেনিয়া তার অফিসে তার বাবাকে ছবিটা দেখিয়েছে বলে বেশ জোরে বলেছে?

2 d সাবরিনা কখন সেনিয়া তার অফিসে তার বাবাকে ছবিটা দেখিয়েছে বলে বেশ জোরে বলেছে?

2 e সাবরিনা কাকে বেশ জোরে বলেছে যে সেনিয়া তার অফিসে তার বাবাকে ছবিটা দেখিয়েছে?

2 f সাবরিনা কখন বেশ জোরে বলেছে যে সেনিয়া তার অফিসে তার বাবাকে ছবিটা দেখিয়েছে?

2 g সাবরিনা কাকে সেনিয়া তার অফিসে ছবিটা দেখিয়েছে বলে বেশ জোরে তার বাবাকে বলেছে?

2 h সাবরিনা কখন সেনিয়া তার অফিসে ছবিটা দেখিয়েছে বলে বেশ জোরে তার বাবাকে বলেছে?

3 a এনাম কাকে কিছুটা বিস্মিতভাবে তার নানাকে বলেছে যে সুমী বাড়িতে ব্যাপারটা বুঝিয়ে দেবে?

3 b এনাম কখন কিছুটা বিস্মিতভাবে তার নানাকে বলেছে যে সুমী বাড়িতে ব্যাপারটা বুঝিয়ে দেবে?

3 c এনাম কাকে সুমী বাড়িতে তার নানাকে ব্যাপারটা বুঝিয়ে দেবে বলে কিছুটা বিস্মিতভাবে বলেছে?

3 d এনাম কখন সুমী বাড়িতে তার নানাকে ব্যাপারটা বুঝিয়ে দেবে বলে কিছুটা বিস্মিতভাবে বলেছে?

3 e এনাম কাকে কিছুটা বিস্মিতভাবে বলেছে যে সুমী বাড়িতে তার নানাকে ব্যাপারটা বুঝিয়ে দেবে?

3 f এনাম কখন কিছুটা বিস্মিতভাবে বলেছে যে সুমী বাড়িতে তার নানাকে ব্যাপারটা বুঝিয়ে দেবে?

3 g এনাম কাকে সুমী বাড়িতে ব্যাপারটা বুঝিয়ে দেবে বলে কিছুটা বিস্মিতভাবে তার নানাকে বলেছে?

3 h এনাম কখন সুমী বাড়িতে ব্যাপারটা বুঝিয়ে দেবে বলে কিছুটা বিস্মিতভাবে তার নানাকে বলেছে?

4 a সালমা কাকে খানিকটা সাবধানে রোকেয়াকে বলেছে যে তানভীর দোকানে বকা দিয়েছে?

4 b সালমা কখন খানিকটা সাবধানে রোকেয়াকে বলেছে যে তানভীর দোকানে বকা দিয়েছে?

4 c সালমা কাকে তানভীর দোকানে রোকেয়াকে বকা দিয়েছে বলে খানিকটা সাবধানে বলেছে?

4 d সালমা কখন তানভীর দোকানে রোকেয়াকে বকা দিয়েছে বলে খানিকটা সাবধানে বলেছে?

4 e সালমা কাকে খানিকটা সাবধানে বলেছে যে তানভীর দোকানে রোকেয়াকে বকা দিয়েছে?

4 f সালমা কখন খানিকটা সাবধানে বলেছে যে তানভীর দোকানে রোকেয়াকে বকা দিয়েছে?

4 g সালমা কাকে তানভীর দোকানে বকা দিয়েছে বলে খানিকটা সাবধানে রোকেয়াকে বলেছে?

4 h সালমা কখন তানভীর দোকানে বকা দিয়েছে বলে খানিকটা সাবধানে রোকেয়াকে বলেছে?

5 a আকাশ কাকে অত্যন্ত সহজভাবে তার মেয়েকে বলেছে যে সোমা ডাকঘরে একটি চিঠি পাঠিয়েছে?

5 b আকাশ কখন অত্যন্ত সহজভাবে তার মেয়েকে বলেছে যে সোমা ডাকঘরে একটি চিঠি পাঠিয়েছে?

5 c আকাশ কাকে সোমা ডাকঘরে তার মেয়েকে একটি চিঠি পাঠিয়েছে বলে অত্যন্ত সহজভাবে বলেছে?

5 d আকাশ কখন সোমা ডাকঘরে তার মেয়েকে একটি চিঠি পাঠিয়েছে বলে অত্যন্ত সহজভাবে বলেছে?

5 e আকাশ কাকে অত্যন্ত সহজভাবে বলেছে যে সোমা ডাকঘরে তার মেয়েকে একটি চিঠি পাঠিয়েছে?

5 f আকাশ কখন অত্যন্ত সহজভাবে বলেছে যে সোমা ডাকঘরে তার মেয়েকে একটি চিঠি পাঠিয়েছে?

5 g আকাশ কাকে সোমা ডাকঘরে একটি চিঠি পাঠিয়েছে বলে অত্যন্ত সহজভাবে তার মেয়েকে বলেছে?

5 h আকাশ কখন সোমা ডাকঘরে একটি চিঠি পাঠিয়েছে বলে অত্যন্ত সহজভাবে তার মেয়েকে বলেছে?

6 a পরশ কাকে অনেকটা সোজাসুজিভাবে জামালকে বলেছে যে রিনা তার স্কুলে পরীক্ষা লিখে দেবে?

6 b পরশ কখন অনেকটা সোজাসুজিভাবে জামালকে বলেছে যে রিনা তার স্কুলে পরীক্ষা লিখে দেবে?

6 c পরশ কাকে রিনা তার স্কুলে জামালকে পরীক্ষা লিখে দেবে বলে অনেকটা সোজাসুজিভাবে বলেছে?

6 d পরশ কখন রিনা তার স্কুলে জামালকে পরীক্ষা লিখে দেবে বলে অনেকটা সোজাসুজিভাবে বলেছে?

6 e পরশ কাকে অনেকটা সোজাসুজিভাবে বলেছে যে রিনা তার স্কুলে জামালকে পরীক্ষা লিখে দেবে?

6 f পরশ কখন অনেকটা সোজাসুজিভাবে বলেছে যে রিনা তার স্কুলে জামালকে পরীক্ষা লিখে দেবে?

6 g পরশ কাকে রিনা তার স্কুলে পরীক্ষা লিখে দেবে বলে অনেকটা সোজাসুজিভাবে জামালকে বলেছে?

6 h পরশ কখন রিনা তার স্কুলে পরীক্ষা লিখে দেবে বলে অনেকটা সোজাসুজিভাবে জামালকে বলেছে?

7 a জয় কাকে কিছুটা বিষণ্নভাবে তার চাচাকে বলেছে যে মিতা রেস্টুরেন্টে চা বানিয়েছে?

7 b জয় কখন কিছুটা বিষণ্নভাবে তার চাচাকে বলেছে যে মিতা রেস্টুরেন্টে চা বানিয়েছে?

7 c জয় কাকে মিতা রেস্টুরেন্টে তার চাচাকে চা বানিয়েছে বলে কিছুটা বিষণ্নভাবে বলেছে?

7 d জয় কখন মিতা রেস্টুরেন্টে তার চাচাকে চা বানিয়েছে বলে কিছুটা বিষণ্নভাবে বলেছে?

7 e জয় কাকে কিছুটা বিষণ্নভাবে বলেছে যে মিতা রেস্টুরেন্টে তার চাচাকে চা বানিয়েছে?
7 f জয় কখন কিছুটা বিষণ্নভাবে বলেছে যে মিতা রেস্টুরেন্টে তার চাচাকে চা বানিয়েছে?

7 g জয় কাকে মিতা রেস্টুরেন্টে চা বানিয়েছে বলে কিছুটা বিষণ্নভাবে তার চাচাকে বলেছে?

7 h জয় কখন মিতা রেস্টুরেন্টে চা বানিয়েছে বলে কিছুটা বিষণ্নভাবে তার চাচাকে বলেছে?

8 a সজল কাকে খুব ধীরে তার চাচীকে বলেছে যে আলম রাস্তায় সিঙ্গারা বানাবে?

8 b সজল কখন খুব ধীরে তার চাচীকে বলেছে যে আলম রাস্তায় সিঙ্গারা বানাবে?

8 c সজল কাকে আলম রাস্তায় তার চাচীকে সিঙ্গারা বানাবে বলে খুব ধীরে বলেছে?

8 d সজল কখন আলম রাস্তায় তার চাচীকে সিঙ্গারা বানাবে বলে খুব ধীরে বলেছে?

8 e সজল কাকে খুব ধীরে বলেছে যে আলম রাস্তায় তার চাচীকে সিঙ্গারা বানাবে?

8 f সজল কখন খুব ধীরে বলেছে যে আলম রাস্তায় তার চাচীকে সিঙ্গারা বানাবে?

8 g সজল কাকে আলম রাস্তায় সিঙ্গারা বানাবে বলে খুব ধীরে তার চাচীকে বলেছে?

8 h সজল কখন আলম রাস্তায় সিঙ্গারা বানাবে বলে খুব ধীরে তার চাচীকে বলেছে?

9 a নয়ন কাকে কিছুটা রহস্যজনকভাবে শান্তাকে বলেছে যে রোকন বাগানে মিথ্যা বলেছে?

9 b নয়ন কখন কিছুটা রহস্যজনকভাবে শান্তাকে বলেছে যে রোকন বাগানে মিথ্যা বলেছে?

9 c নয়ন কাকে রোকন বাগানে শান্তাকে মিথ্যা বলেছে বলে কিছুটা রহস্যজনকভাবে বলেছে?

9 d নয়ন কখন রোকন বাগানে শান্তাকে মিথ্যা বলেছে বলে কিছুটা রহস্যজনকভাবে বলেছে?

9 e নয়ন কাকে কিছুটা রহস্যজনকভাবে বলেছে যে রোকন বাগানে শান্তাকে মিথ্যা বলেছে?

9 f নয়ন কখন কিছুটা রহস্যজনকভাবে বলেছে যে রোকন বাগানে শান্তাকে মিথ্যা বলেছে?

9 g নয়ন কাকে রোকন বাগানে মিথ্যা বলেছে বলে কিছুটা রহস্যজনকভাবে শান্তাকে বলেছে?

9 h নয়ন কখন রোকন বাগানে মিথ্যা বলেছে বলে কিছুটা রহস্যজনকভাবে শান্তাকে বলেছে?

10 a শীলা কাকে বেশ তাড়াতাড়ি দোকানদারকে বলেছে যে মণীষা শপিংমলে অপমান করেছে?

10 b শীলা কখন বেশ তাড়াতাড়ি দোকানদারকে বলেছে যে মণীষা শপিংমলে অপমান করেছে?

10 c শীলা কাকে মণীষা শপিংমলে দোকানদারকে অপমান করেছে বলে বেশ তাড়াতাড়ি বলেছে?

10 d শীলা কখন মণীষা শপিংমলে দোকানদারকে অপমান করেছে বলে বেশ তাড়াতাড়ি বলেছে?

10 e শীলা কাকে বেশ তাড়াতাড়ি বলেছে যে মণীষা শপিংমলে দোকানদারকে অপমান করেছে?

10 f শীলা কখন বেশ তাড়াতাড়ি বলেছে যে মণীষা শপিংমলে দোকানদারকে অপমান করেছে?

10 g শীলা কাকে মণীষা শপিংমলে অপমান করেছে বলে বেশ তাড়াতাড়ি দোকানদারকে বলেছে?

10 h শীলা কখন মণীষা শপিংমলে অপমান করেছে বলে বেশ তাড়াতাড়ি দোকানদারকে বলেছে?

11 a সায়েম কাকে খুব ভদ্রভাবে প্রতিবেশীকে বলেছে যে নাজিয়া বাজারে স্বাগত জানিয়েছে?

11 b সায়েম কখন খুব ভদ্রভাবে প্রতিবেশীকে বলেছে যে নাজিয়া বাজারে স্বাগত জানিয়েছে?

11 c সায়েম কাকে নাজিয়া বাজারে প্রতিবেশীকে স্বাগত জানিয়েছে বলে খুব ভদ্রভাবে বলেছে?

11 d সায়েম কখন নাজিয়া বাজারে প্রতিবেশীকে স্বাগত জানিয়েছে বলে খুব ভদ্রভাবে বলেছে?

11 e সায়েম কাকে খুব ভদ্রভাবে বলেছে যে নাজিয়া বাজারে প্রতিবেশীকে স্বাগত জানিয়েছে?

11 f সায়েম কখন খুব ভদ্রভাবে বলেছে যে নাজিয়া বাজারে প্রতিবেশীকে স্বাগত জানিয়েছে?

11 g সায়েম কাকে নাজিয়া বাজারে স্বাগত জানিয়েছে বলে খুব ভদ্রভাবে প্রতিবেশীকে বলেছে?

11 h সায়েম কখন নাজিয়া বাজারে স্বাগত জানিয়েছে বলে খুব ভদ্রভাবে প্রতিবেশীকে বলেছে?

12 a রূপা কাকে বেশ খানিকটা সাবধানে ললিতাকে বলেছে যে রমেন বাইরে ইংরেজি পড়িয়েছে?

12 b রূপা কখন বেশ খানিকটা সাবধানে ললিতাকে বলেছে যে রমেন বাইরে ইংরেজি পড়িয়েছে?

12 c রূপা কাকে রমেন বাইরে ললিতাকে ইংরেজি পড়িয়েছে বলে বেশ খানিকটা সাবধানে বলেছে?

12 d রূপা কখন রমেন বাইরে ললিতাকে ইংরেজি পড়িয়েছে বলে বেশ খানিকটা সাবধানে বলেছে?

12 e রূপা কাকে বেশ খানিকটা সাবধানে বলেছে যে রমেন বাইরে ললিতাকে ইংরেজি পড়িয়েছে?

12 f রূপা কখন বেশ খানিকটা সাবধানে বলেছে যে রমেন বাইরে ললিতাকে ইংরেজি পড়িয়েছে?

12 g রূপা কাকে রমেন বাইরে ইংরেজি পড়িয়েছে বলে বেশ খানিকটা সাবধানে ললিতাকে বলেছে?

12 h রূপা কখন রমেন বাইরে ইংরেজি পড়িয়েছে বলে বেশ খানিকটা সাবধানে ললিতাকে বলেছে?

13 a ইভা কাকে অনেকটা গোপনে তার মাকে বলেছে যে করিম আটটায় ফোন করেছে?

13 b ইভা কোথায় অনেকটা গোপনে তার মাকে বলেছে যে করিম আটটায় ফোন করেছে?

13 c ইভা কাকে করিম আটটায় তার মাকে ফোন করেছে বলে অনেকটা গোপনে বলেছে?

13 d ইভা কোথায় করিম আটটায় তার মাকে ফোন করেছে বলে অনেকটা গোপনে বলেছে?

13 e ইভা কাকে অনেকটা গোপনে বলেছে যে করিম আটটায় তার মাকে ফোন করেছে?

13 f ইভা কোথায় অনেকটা গোপনে বলেছে যে করিম আটটায় তার মাকে ফোন করেছে?

13 g ইভা কাকে করিম আটটায় ফোন করেছে বলে অনেকটা গোপনে তার মাকে বলেছে?

13 h ইভা কোথায় করিম আটটায় ফোন করেছে বলে অনেকটা গোপনে তার মাকে বলেছে?

14 a রাশাদ কাকে বেশ শান্তভাবে তার বান্ধবীকে বলেছে যে সামীরা তিনটায় গণিত শিখিয়েছে?

14 b রাশাদ কোথায় বেশ শান্তভাবে তার বান্ধবীকে বলেছে যে সামীরা তিনটায় গণিত শিখিয়েছে?

14 c রাশাদ কাকে সামীরা তিনটায় তার বান্ধবীকে গণিত শিখিয়েছে বলে বেশ শান্তভাবে বলেছে?

14 d রাশাদ কোথায় সামীরা তিনটায় তার বান্ধবীকে গণিত শিখিয়েছে বলে বেশ শান্তভাবে বলেছে?

14 e রাশাদ কাকে বেশ শান্তভাবে বলেছে যে সামীরা তিনটায় তার বান্ধবীকে গণিত শিখিয়েছে?

14 f রাশাদ কোথায় বেশ শান্তভাবে বলেছে যে সামীরা তিনটায় তার বান্ধবীকে গণিত শিখিয়েছে?

14 g রাশাদ কাকে সামীরা তিনটায় গণিত শিখিয়েছে বলে বেশ শান্তভাবে তার বান্ধবীকে বলেছে?

14 h রাশাদ কোথায় সামীরা তিনটায় গণিত শিখিয়েছে বলে বেশ শান্তভাবে তার বান্ধবীকে বলেছে?

15 a মনির কাকে কিছুটা রাগতভাবে তার ভাইকে বলেছে যে অজয় দুটোয় কাহিনী শুনিয়েছে?

15 b মনির কোথায় কিছুটা রাগতভাবে তার ভাইকে বলেছে যে অজয় দুটোয় কাহিনী শুনিয়েছে?

15 c মনির কাকে অজয় দুটোয় তার ভাইকে কাহিনী শুনিয়েছে বলে কিছুটা রাগতভাবে বলেছে?

15 d মনির কোথায় অজয় দুটোয় তার ভাইকে কাহিনী শুনিয়েছে বলে কিছুটা রাগতভাবে বলেছে?

15 e মনির কাকে কিছুটা রাগতভাবে বলেছে যে অজয় দুটোয় তার ভাইকে কাহিনী শুনিয়েছে?

15 f মনির কোথায় কিছুটা রাগতভাবে বলেছে যে অজয় দুটোয় তার ভাইকে কাহিনী শুনিয়েছে?

15 g মনির কাকে অজয় দুটোয় কাহিনী শুনিয়েছে বলে কিছুটা রাগতভাবে তার ভাইকে বলেছে?

15 h মনির কোথায় অজয় দুটোয় কাহিনী শুনিয়েছে বলে কিছুটা রাগতভাবে তার ভাইকে বলেছে?

16 a জাহিদ কাকে প্রায় উত্তেজিতভাবে তার বোনকে বলেছে যে নায়রা দুপুরে একটি বই দিয়েছে?

16 b জাহিদ কোথায় প্রায় উত্তেজিতভাবে তার বোনকে বলেছে যে নায়রা দুপুরে একটি বই দিয়েছে?

16 c জাহিদ কাকে নায়রা দুপুরে তার বোনকে একটি বই দিয়েছে বলে প্রায় উত্তেজিতভাবে বলেছে?

16 d জাহিদ কোথায় নায়রা দুপুরে তার বোনকে একটি বই দিয়েছে বলে প্রায় উত্তেজিতভাবে বলেছে?

16 e জাহিদ কাকে প্রায় উত্তেজিতভাবে বলেছে যে নায়রা দুপুরে তার বোনকে একটি বই দিয়েছে?

16 f জাহিদ কোথায় প্রায় উত্তেজিতভাবে বলেছে যে নায়রা দুপুরে তার বোনকে একটি বই দিয়েছে?

16 g জাহিদ কাকে নায়রা দুপুরে একটি বই দিয়েছে বলে প্রায় উত্তেজিতভাবে তার বোনকে বলেছে?

16 h জাহিদ কোথায় নায়রা দুপুরে একটি বই দিয়েছে বলে প্রায় উত্তেজিতভাবে তার বোনকে বলেছে?

17 a আহমেদ কাকে বেশ গর্বিতভাবে সুজনকে বলেছে যে রাজ বারোটায় গল্প শুনিয়েছে?

17 b আহমেদ কোথায় বেশ গর্বিতভাবে সুজনকে বলেছে যে রাজ বারোটায় গল্প শুনিয়েছে?

17 c আহমেদ কাকে রাজ বারোটায় সুজনকে গল্প শুনিয়েছে বলে বেশ গর্বিতভাবে বলেছে?

17 d আহমেদ কোথায় রাজ বারোটায় সুজনকে গল্প শুনিয়েছে বলে বেশ গর্বিতভাবে বলেছে?

17 e আহমেদ কাকে বেশ গর্বিতভাবে বলেছে যে রাজ বারোটায় সুজনকে গল্প শুনিয়েছে?

17 f আহমেদ কোথায় বেশ গর্বিতভাবে বলেছে যে রাজ বারোটায় সুজনকে গল্প শুনিয়েছে?

17 g আহমেদ কাকে রাজ বারোটায় গল্প শুনিয়েছে বলে বেশ গর্বিতভাবে সুজনকে বলেছে?

17 h আহমেদ কোথায় রাজ বারোটায় গল্প শুনিয়েছে বলে বেশ গর্বিতভাবে সুজনকে বলেছে?

18 a রজানা কাকে খুব সহজভাবে সাদাতকে বলেছে যে মৌরি বৃহস্পতিবারে একটি কলম দিয়েছে?

18 b ফারজানা কোথায় খুব সহজভাবে সাদাতকে বলেছে যে মৌরি বৃহস্পতিবারে একটি কলম দিয়েছে?

18 c ফারজানা কাকে মৌরি বৃহস্পতিবারে সাদাতকে একটি কলম দিয়েছে বলে খুব সহজভাবে বলেছে?

18 d ফারজানা কোথায় মৌরি বৃহস্পতিবারে সাদাতকে একটি কলম দিয়েছে বলে খুব সহজভাবে বলেছে?

18 e ফারজানা কাকে খুব সহজভাবে বলেছে যে মৌরি বৃহস্পতিবারে সাদাতকে একটি কলম দিয়েছে?

18 f ফারজানা কোথায় খুব সহজভাবে বলেছে যে মৌরি বৃহস্পতিবারে সাদাতকে একটি কলম দিয়েছে?

18 g ফারজানা কাকে মৌরি বৃহস্পতিবারে একটি কলম দিয়েছে বলে খুব সহজভাবে সাদাতকে বলেছে?

18 h ফারজানা কোথায় মৌরি বৃহস্পতিবারে একটি কলম দিয়েছে বলে খুব সহজভাবে সাদাতকে বলেছে?

19 a অর্ণব কাকে কিছুটা কৌতূহলীভাবে সোহানকে বলেছে যে প্রিয়ান্কা এগারোটায় সিনেমা দেখিয়েছে?

19 b অর্ণব কোথায় কিছুটা কৌতূহলীভাবে সোহানকে বলেছে যে প্রিয়ান্কা এগারোটায় সিনেমা দেখিয়েছে?

19 c অর্ণব কাকে প্রিয়ান্কা এগারোটায় সোহানকে সিনেমা দেখিয়েছে বলে কিছুটা কৌতূহলীভাবে বলেছে?

19 d অর্ণব কোথায় প্রিয়ান্কা এগারোটায় সোহানকে সিনেমা দেখিয়েছে বলে কিছুটা কৌতূহলীভাবে বলেছে?

19 e অর্ণব কাকে কিছুটা কৌতূহলীভাবে বলেছে যে প্রিয়ান্‌কা এগারোটায় সোহানকে সিনেমা দেখিয়েছে?

19 f অর্ণব কোথায় কিছুটা কৌতূহলীভাবে বলেছে যে প্রিয়ান্‌কা এগারোটায় সোহানকে সিনেমা দেখিয়েছে?

19 g অর্ণব কাকে প্রিয়ান্‌কা এগারোটায় সিনেমা দেখিয়েছে বলে কিছুটা কৌতূহলীভাবে সোহানকে বলেছে?

19 h অর্ণব কোথায় প্রিয়ান্‌কা এগারোটায় সিনেমা দেখিয়েছে বলে কিছুটা কৌতূহলীভাবে সোহানকে বলেছে?

20 a আতাউর কাকে খুব গোপনে শাহনাজকে বলেছে যে নাসরিন মঙ্গলবারে আভিবাদন জানিয়েছে?

20 b আতাউর কোথায় খুব গোপনে শাহনাজকে বলেছে যে নাসরিন মঙ্গলবারে আভিবাদন জানিয়েছে?

20 c আতাউর কাকে নাসরিন মঙ্গলবারে শাহনাজকে আভিবাদন জানিয়েছে বলে খুব গোপনে বলেছে?

20 d আতাউর কোথায় নাসরিন মঙ্গলবারে শাহনাজকে আভিবাদন জানিয়েছে বলে খুব গোপনে বলেছে?

20 e আতাউর কাকে খুব গোপনে বলেছে যে নাসরিন মঙ্গলবারে শাহনাজকে আভিবাদন জানিয়েছে?

20 f আতাউর কোথায় খুব গোপনে বলেছে যে নাসরিন মঙ্গলবারে শাহনাজকে আভিবাদন জানিয়েছে?

20 g আতাউর কাকে নাসরিন মঙ্গলবারে আভিবাদন জানিয়েছে বলে খুব গোপনে শাহনাজকে বলেছে?

20 h আতাউর কোথায় নাসরিন মঙ্গলবারে আভিবাদন জানিয়েছে বলে খুব গোপনে শাহনাজকে বলেছে?

21 a অরুণ কাকে প্রায় নিখুঁতভাবে তার মামাকে বলেছে যে সংগীতা তার জন্মদিনে সাহায্য করেছে?

21 b অরুণ কোথায় প্রায় নিখুঁতভাবে তার মামাকে বলেছে যে সংগীতা তার জন্মদিনে সাহায্য করেছে?

21 c অরুণ কাকে সংগীতা তার জন্মদিনে তার মামাকে সাহায্য করেছে বলে প্রায় নিখুঁতভাবে বলেছে?

21 d অরুণ কোথায় সংগীতা তার জন্মদিনে তার মামাকে সাহায্য করেছে বলে প্রায় নিখুঁতভাবে বলেছে?

21 e অরুণ কাকে প্রায় নিখুঁতভাবে বলেছে যে সংগীতা তার জন্মদিনে তার মামাকে সাহায্য করেছে?

21 f অরুণ কোথায় প্রায় নিখুঁতভাবে বলেছে যে সংগীতা তার জন্মদিনে তার মামাকে সাহায্য করেছে?

21 g অরুণ কাকে সংগীতা তার জন্মদিনে সাহায্য করেছে বলে প্রায় নিখুঁতভাবে তার মামাকে বলেছে?

21 h অরুণ কোথায় সংগীতা তার জন্মদিনে সাহায্য করেছে বলে প্রায় নিখুঁতভাবে তার মামাকে বলেছে?

22 a গীতালি কাকে বেশ স্পষ্টভাবে তার খালাকে বলেছে যে মিলি সকালে ঠোকিয়েছে?

22 b গীতালি কোথায় বেশ স্পষ্টভাবে তার খালাকে বলেছে যে মিলি সকালে ঠোকিয়েছে?

22 c গীতালি কাকে মিলি সকালে তার খালাকে ঠোকিয়েছে বলে বেশ স্পষ্টভাবে বলেছে?

22 d গীতালি কোথায় মিলি সকালে তার খালাকে ঠোকিয়েছে বলে বেশ স্পষ্টভাবে বলেছে?

22 e গীতালি কাকে বেশ স্পষ্টভাবে বলেছে যে মিলি সকালে তার খালাকে ঠোকিয়েছে?

22 f গীতালি কোথায় বেশ স্পষ্টভাবে বলেছে যে মিলি সকালে তার খালাকে ঠোকিয়েছে?

22 g গীতালি কাকে মিলি সকালে ঠোকিয়েছে বলে বেশ স্পষ্টভাবে তার খালাকে বলেছে?

22 h গীতালি কোথায় মিলি সকালে ঠোকিয়েছে বলে বেশ স্পষ্টভাবে তার খালাকে বলেছে?

23 a লিপি কাকে অনেক জোরালোভাবে শিক্ষকটিকে বলেছে যে হীরা ছুটির সময়ে অপমান করেছে?

23 b লিপি কোথায় অনেক জোরালোভাবে শিক্ষকটিকে বলেছে যে হীরা ছুটির সময়ে অপমান করেছে?

23 c লিপি কাকে হীরা ছুটির সময়ে শিক্ষকটিকে অপমান করেছে বলে অনেক জোরালোভাবে বলেছে?

23 d লিপি কোথায় হীরা ছুটির সময়ে শিক্ষকটিকে অপমান করেছে বলে অনেক জোরালোভাবে বলেছে?

23 e লিপি কাকে অনেক জোরালোভাবে বলেছে যে হীরা ছুটির সময়ে শিক্ষকটিকে অপমান করেছে?

23 f লিপি কোথায় অনেক জোরালোভাবে বলেছে যে হীরা ছুটির সময়ে শিক্ষকটিকে অপমান করেছে?

23 g লিপি কাকে হীরা ছুটির সময়ে অপমান করেছে বলে অনেক জোরালোভাবে শিক্ষকটিকে বলেছে?

23 h লিপি কোথায় হীরা ছুটির সময়ে অপমান করেছে বলে অনেক জোরালোভাবে শিক্ষকটিকে বলেছে?

24 a অদিতি কাকে প্রায় নিশ্চিতভাবে ডাক্তারটিকে বলেছে যে ইন্দিরা রাত্রিতে একটা উপহার দিয়েছে?

24 b অদিতি কোথায় প্রায় নিশ্চিতভাবে ডাক্তারটিকে বলেছে যে ইন্দিরা রাত্রিতে একটা উপহার দিয়েছে?

24 c অদিতি কাকে ইন্দিরা রাত্রিতে ডাক্তারটিকে একটা উপহার দিয়েছে বলে প্রায় নিশ্চিতভাবে বলেছে?

24 d অদিতি কোথায় ইন্দিরা রাত্রিতে ডাক্তারটিকে একটা উপহার দিয়েছে বলে প্রায় নিশ্চিতভাবে বলেছে?

24 e অদিতি কাকে প্রায় নিশ্চিতভাবে বলেছে যে ইন্দিরা রাত্রিতে ডাক্তারটিকে একটা উপহার দিয়েছে?

24 f অদিতি কোথায় প্রায় নিশ্চিতভাবে বলেছে যে ইন্দিরা রাত্রিতে ডাক্তারটিকে একটা উপহার দিয়েছে?

24 g অদিতি কাকে ইন্দিরা রাত্রিতে একটা উপহার দিয়েছে বলে প্রায় নিশ্চিতভাবে ডাক্তারটিকে বলেছে?

24 h অদিতি কোথায় ইন্দিরা রাত্রিতে একটা উপহার দিয়েছে বলে প্রায় নিশ্চিতভাবে ডাক্তারটিকে বলেছে?
